# Discovering interpretable drug formulation behavior patterns via a mechanistic-augmented conditional variational autoencoder

**DOI:** 10.1038/s41598-026-61464-z

**Published:** 2026-07-15

**Authors:** El-Sayed Khafagy, Amr Selim Abu Lila, Ahmed Al Saqr, Mahboubeh Pishnamazi

**Affiliations:** 1 Department of Pharmaceutics, College of Pharmacy, Prince Sattam Bin Abdulaziz University, 11942, Al-Kharj, Saudi Arabia; 2 Department of Pharmaceutics, College of Pharmacy, University of Ha’il, 81442 Ha’il, Saudi Arabia; 3 Institute of Research and Development, Duy Tan University, Da Nang, Vietnam; 4 School of Engineering and Technology, Duy Tan University, Da Nang, Vietnam

**Keywords:** Drug formulation behaviour, Solubility modelling, Particle size, Latent space representation, Chemistry, Computational biology and bioinformatics, Materials science, Mathematics and computing

## Abstract

Formulation composition, processing conditions, and their combined effect on drug solubility and particle size are not fully understood. This is due to a complicated network of interactions dependent on the conditions, which are usually investigated by trial-and-error testing. A question that has been left practically unanswered is whether the experimental formulation data can still find patterns of structured and interpretable behavior that are beyond mere prediction. This paper tries to answer whether the latent generative modeling can structurize formulation knowledge in a continuous, regime-aware manner. 114 niosome formulation samples were mined systematically from 17 publications based on the PRISMA framework. Inputs to model the encapsulated drug efficiency and particle size were 11 drug, formulation, and processing variables. To develop a structured latent understanding of formulation behavior, a mechanistic-augmented conditional variational autoencoder was used. The discovered latent space became a continuum with regimes overlapping, smooth changes, and feature, response relationships being dependent on the context. In a sense, formulation behavior can be considered as a structured latent landscape that can be used for regime-aware analysis and the generation of hypotheses in data-driven formulation research.

**Supplementary Information:**

The online version contains supplementary material available at 10.1038/s41598-026-61464-z.

## Introduction

The discovery and development of successful pharmaceutical products have traditionally depended on significant trial-and-error testing, a method that is time-consuming, costly, and frequently inefficient^1,2^. In recent years, ML has become an increasingly essential technique in drug discovery, giving data-driven ways to predict complicated interactions and minimize experimental load^3^. While early ML applications have largely focused on molecular discovery and optimization, similar challenges persist in drug formulation development, where critical performance attributes such as solubility and particle size emerge from nonlinear interactions between formulation composition and processing conditions^4,5^. These complexities stimulate the need for models that may transcend beyond prediction and reveal underlying formulation behavior patterns. Generative models have attracted great interest in this domain because of their capacity to learn compact, low-dimensional representations of high-dimensional data^6,7^. Among these models, VAEs provide a systematic framework for learning continuous latent spaces using an encoder-decoder architecture^8^. By mapping observed data into a structured latent representation and reconstructing it back to the original space, VAEs may capture smooth transitions in the underlying data distribution^9–11^. In formulation modeling, such latent spaces have the possibility to represent continuous transitions between formulation regimes, rather than considering formulations as isolated experimental points, permitting systematic study of formulation behavior^12^.

Despite these improvements, a significant restriction of typical VAEs resides in the entanglement of their hidden representations. It is challenging to comprehend how changes in latent variables relate to changes in observable qualities in highly entangled latent spaces since individual dimensions do not correlate to independent or interpretable aspects^13^. Previous research in interpretable machine learning has put forth a number of methods to increase generative modeling transparency, frequently focusing on stepwise building procedures or structural similarities. However, these approaches typically focus on interpretability with respect to structural features, leaving a critical gap in property-driven latent space interpretability, where latent variables are expected to correspond to meaningful variations in physicochemical behavior rather than structural resemblance alone. To solve this difficulty, numerous adaptations of the original “vanilla” VAE architecture have been proposed. In the conventional formulation, VAEs optimize a loss function comprised of a reconstruction term and a KL divergence term, which regularizes the latent space to follow a prior distribution^14^. Although this approach works well for generative tasks, it frequently fails to generate disentangled latent representations. By explicitly promoting factorized and disentangled latent dimensions, the β-VAE alters the relative weight of the KL divergence term. Such disentanglement is particularly important in formulation investigations, where individual latent dimensions may correspond to physically significant parameters impacting solubility, particle size, or their smooth transitions under changing process conditions. Compared with simply data-driven embeddings, disentangled latent spaces give a firmer platform for interpretation and scientific insight^15^.

Latent spaces facilitate the discovery of structure in formulation datasets in addition to representation learning. By evaluating the geometry of the learnt latent representations, formulations may be divided into clusters according to unique formulation regimes or behavior patterns^16^. When paired with dimensionality reduction, clustering, and post-hoc interpretability techniques, latent representations can serve as a bridge between sophisticated ML models and mechanistic knowledge^17^. This viewpoint moves the function of generative models from black-box generators toward interpretable frameworks capable of exposing formulation behavior patterns and aiding rational formulation design.

VAEs and CVAEs have become popular tools in chemical and pharmaceutical research for obtaining compact latent representations of complex data. The majority of applications are still structure-centric, e.g., generating molecules, exploring diversity, and designing property-conditioned molecules. Ochiai et al.^18^ illustrated how VAEs efficiently disentangle complicated 3D molecular structures and organize them into latent manifolds that are coherent and easy to navigate, whereas Janela et al.^19^ used CVAEs conditioned on potency fingerprints for molecule prediction. Yue et al.^20^ took this one step further by proposing an interpretable CVAE with a controllable mapping between latent variables and molecular properties. While these works demonstrated that generative latent models are capable of encoding chemically meaningful information, it remains the case that the latent space serves more as a means for structure exploration or prediction rather than being a topic of scientific investigation. As the applications of ML in pharmaceutics moved beyond the scope of molecular discovery, predicting properties such as solubility under different formulation conditions became the focus. Ghanavati et al.^21^ combined GCNs and XGBoost to predict aqueous solubility from physicochemical descriptors and used SHAP for post-hoc explanation of the model. Works related to this one have attempted the modeling of solubility in binary solvent mixtures at different temperatures^22^ and have used GCNs to capture the nonlinear solvent–temperature–solubility relationship^23^. These publications highlight the significance of condition-dependent property modeling and explainable ML. Still, the issue of interpretability here is mostly limited to justifying predictions at the sample level rather than gaining insight into how the learned representation itself is structured. Another line of research complements the above by investigating how latent spaces can be structured for interpretability. Zeng et al.^24^ found that disentangled VAEs can isolate target properties from confounders in materials design, thus resulting in interpretable latent dimensions. Fan et al.^25^ introduced a Gaussian Mixture VAE in which latent clusters correspond to physically meaningful regimes and help stabilize training. Hasankhani^26^ showed how solubility prediction is improved by latent-space interpolation guided by clustering, while the PCF-VAE model^27^ tackled posterior collapse to guarantee latent variables with content. These studies emphasize that latent organization, disentanglement, and clustering are key ingredients for meaningful representations.

This‍‌ research focuses on the problem of insufficient knowledge regarding interpretable formulation behavior patterns that result from the interaction of process variables and physicochemical properties such as solubility and particle size. Most of the approaches from the past that have been structure-centric or prediction-oriented fail to address a major issue in this work: the latent organization of formulation data is treated as a scientific object that can be used to uncover continuous formulation regimes and transitions induced by temperature and solvent composition. In other words, the formulation data is organized in a way that allows physically consistent patterns to be discovered by a mechanistically guided conditional latent modeling framework, which is at the same time allowed to preserve the disentangled, interpretable structure of the formulation behavior patterns. The latent space that is generated thus captures formulation regime structure, allows the continuous behavioral manifolds to be visualized, and clusters the formulation patterns. Hence, the formulated sample-level prediction is considered as scientifically reasonable, and it opens the door for formulation understanding, hypothesis generation, and design ‍‌guidance.

## Dataset description and curation

The dataset for the present study was generated by a thorough manual extraction of experimental formulation data from peer-reviewed articles on vesicular drug delivery systems after conducting a PRISMA-guided screening procedure. The entire curated dataset is available in^28^ and contains 114 independent formulation samples taken from studies with reports of both drug solubility in water and vesicular particle size prepared under controlled hydration methods^28–45^. Studies were included only if they met the criteria: (i) the molecular descriptors, formulation parameters, and processing conditions were numerically reported in full; (ii) both solubility and particle size were quantitatively determined; and (iii) the hydration procedures were similar enough to exclude procedural confounding. Clinical and pharmacokinetic studies were omitted to make sure that the dataset represents only in vitro physicochemical formulation behavior.

### Input features and target outputs

Every formulation sample can be characterized by molecular descriptors (MW, LogP, pKa, intrinsic solubility), formulation composition variables (hydration media amount, solvent amount, HLB, drug/lipid ratio, Cholesterol/Span60 ratio), and processing conditions (hydration time and temperature). Two target outputs were measured: aqueous drug solubility (mg·mL⁻^1^) and average vesicle particle size (nm) (Table 1).

**Table 1:** Summary of dataset input features and output variables.

Category	Feature	Symbol	Unit	Description	Functional Role
Molecular Descriptors	Molecular weight	MW	g·mol⁻^1^	Drug molecular mass	Influences diffusion and dissolution behavior
	Lipophilicity	LogP	–	Octanol–water partition coefficient	Governs membrane affinity and solubility
	Ionization constant (acid)	pKa	–	Acid dissociation constant	Determines ionization and aqueous solubility
	Intrinsic solubility	S₀	mg·mL⁻^1^	Solubility in pure water	Baseline solubility reference
Formulation Parameters	Hydration media amount	V_hyd	mL	Volume of aqueous phase	Affects vesicle formation and dilution
	Solvent amount	V_sol	mL	Organic solvent volume	Influences lipid film formation
	Hydrophilic–lipophilic balance	HLB	–	Surfactant polarity index	Controls vesicle stability
	Drug/Lipid ratio	D/L	–	Drug loading fraction	Regulates encapsulation and release
	Cholesterol/Span60 ratio	Chol/S60	–	Membrane rigidity indicator	Affects vesicle integrity
Processing Conditions	Hydration time	t_hyd	min	Duration of hydration	Controls vesicle maturation
	Hydration temperature	T_hyd	°C	Temperature during hydration	Governs lipid fluidity and solubility transition
Target Outputs	Solubility in water	S	mg·mL⁻^1^	Measured aqueous solubility	Primary formulation response
	Particle size	D_p	nm	Mean vesicle diameter	Critical quality attribute

### Literature screening and PRISMA workflow

A PRISMA-guided screening strategy was followed to systematically identify studies reporting experimentally characterized niosomal formulations prepared using thin-film hydration methods. Records were collected from peer-reviewed pharmaceutical and formulation studies indexed in major scientific databases. Duplicate records were removed prior to screening. Titles and abstracts were initially screened to eliminate unrelated studies, followed by full-text eligibility assessment. Studies were included only if:

(i) formulation variables and molecular descriptors were numerically reported,
(ii) particle size and/or solubility measurements were quantitatively available, and
(iii) compatible hydration-based preparation procedures were used.

Studies lacking complete formulation information, containing exclusively in vivo/pharmacokinetic analyses, or employing substantially different preparation methodologies were excluded. A total of 114 formulation samples extracted from 17 eligible studies were retained for latent-space modeling and analysis. Because the dataset was aggregated from independent experimental studies, heterogeneity in formulation composition, measurement conditions, and reporting protocols was expected. To reduce procedural confounding, only studies employing comparable thin-film hydration methodologies were retained. Numerical features were standardized prior to modeling to minimize scale-related variability across studies.

Rather than assuming complete experimental uniformity, the proposed latent modeling framework was designed to accommodate heterogeneous formulation observations and organize them into continuous behavioral regimes. Accordingly, latent clustering and probabilistic representation learning were used to identify shared physicochemical patterns across partially overlapping experimental conditions.

### Dataset characteristics and partitioning

The‍‌ dataset covers a diverse range of drugs, solvent types, levels of hydration, and formulation ratios that have been independently reported in different studies (114 formulation samples). Such diversity allows the discovery of generalizable formulation behavior patterns and not only drug-specific ‍‌trends. Samples at the formulation level were split into training (80%) (91 samples) and testing (20%) (23 samples) sets. The test set was kept completely independent and was not touched during model training or latent space construction to prevent information leakage. The data corresponds to real-life experimental observations of formulation studies and is meant to disclose formulation behavior patterns as well as to serve as a guide for experimental design.

### Data preprocessing and latent space analysis preparation

Before modeling, numerical variables were standardized to have zero mean and unit variance to avoid one variable having more impact than others through latent learning. The Shapiro–Wilk test was used to examine the normality of each variable and guide appropriate scaling based on the distribution of the variables. By comparing learned latent representations geometrically, UMAP was mainly used for dimension reduction to plot latent structuring, along with t-SNE used as a secondary sensitivity check. Latent samples were mathematically clustered by GMM to discover formulation regimes, and HDBSCAN was utilized for density-based clustering as a cluster stability verification. To understand the physicochemical composition of latent clusters, the PDPs were created for each cluster. Additionally, SHAP analysis was run to measure the contribution of variables to solubility and particle size variation within the identified regimes. The whole process of preprocessing and analysis makes it possible to treat latent representations as structured formulation landscapes rather than just abstract embeddings.

Particle-size and solubility measurements exhibited strong right-skewed distributions spanning multiple numerical scales. To reduce scale imbalance and stabilize optimization dynamics, logarithmic transformation was applied prior to model training. This preprocessing step was performed to improve numerical conditioning and variance stabilization rather than to enforce strict Gaussian behavior. The hydrophilic–lipophilic balance (HLB) feature exhibited minimal variation across the retained formulation studies. Consequently, its contribution to latent discrimination was limited relative to other physicochemical and process variables. The feature was retained primarily for formulation completeness and consistency with the extracted literature descriptors rather than for dominant predictive influence.

Table 2 summarizes how formulation samples are distributed in the latent space learned by the mechanistic-augmented C-VAE. The statistics characterize the geometric structure of the embedding rather than model performance. The UMAP and t-SNE projections provided exploratory visualization of the learned latent organization under selected hyperparameter settings. Because projection geometry depends on dimensionality-reduction parameters and random initialization, these visualizations were interpreted qualitatively rather than as definitive quantitative evidence of latent structure. GMM assigns samples into multiple components (0–6), indicating overlapping formulation regimes. Across multiple HDBSCAN parameter settings, strong density-separated clusters were not consistently identified, suggesting that the latent organization may contain partially overlapping formulation regions rather than sharply isolated groups. However, this observation was interpreted cautiously because density-based clustering behavior depends on parameter selection and sample distribution. Under the evaluated HDBSCAN parameter settings, strong density-separated clusters were not consistently identified, and many samples were classified as low-density points. This behavior suggests that the latent representation may contain gradual and overlapping formulation organization rather than sharply isolated density-defined regimes. Because density-based clustering outcomes depend strongly on parameter selection and sample distribution, the observed organization was interpreted cautiously as exploratory evidence of continuous latent structure rather than definitive cluster discovery. Finally, the similarity in scale between real and predicted particle-size values serves as a reconstruction sanity check for the latent representation.

**Table 2:** Latent space structure and cluster behaviour revealed by UMAP/t-SNE projections and model prediction consistency.

	Min. (nm)	Max. (nm)	Average (nm)
Cluster gmm	0	6	3.1404
Cluster HDBSCAN	− 1	− 1	− 1
Particle size real	80.35	59,120	4024.59
Particle size pred	207.12	46,318.79	3807.57
error	− 14,980.80	6421.11	− 217.02
TSNE 1	− 17.07	− 1.35	− 9.48
TSNE 2	− 6.72	5.93	− 0.41
UMAP 1	− 3.42	8.01	0.84
UMAP 2	− 3.43	10.03	3.56

**Fig. 1:**
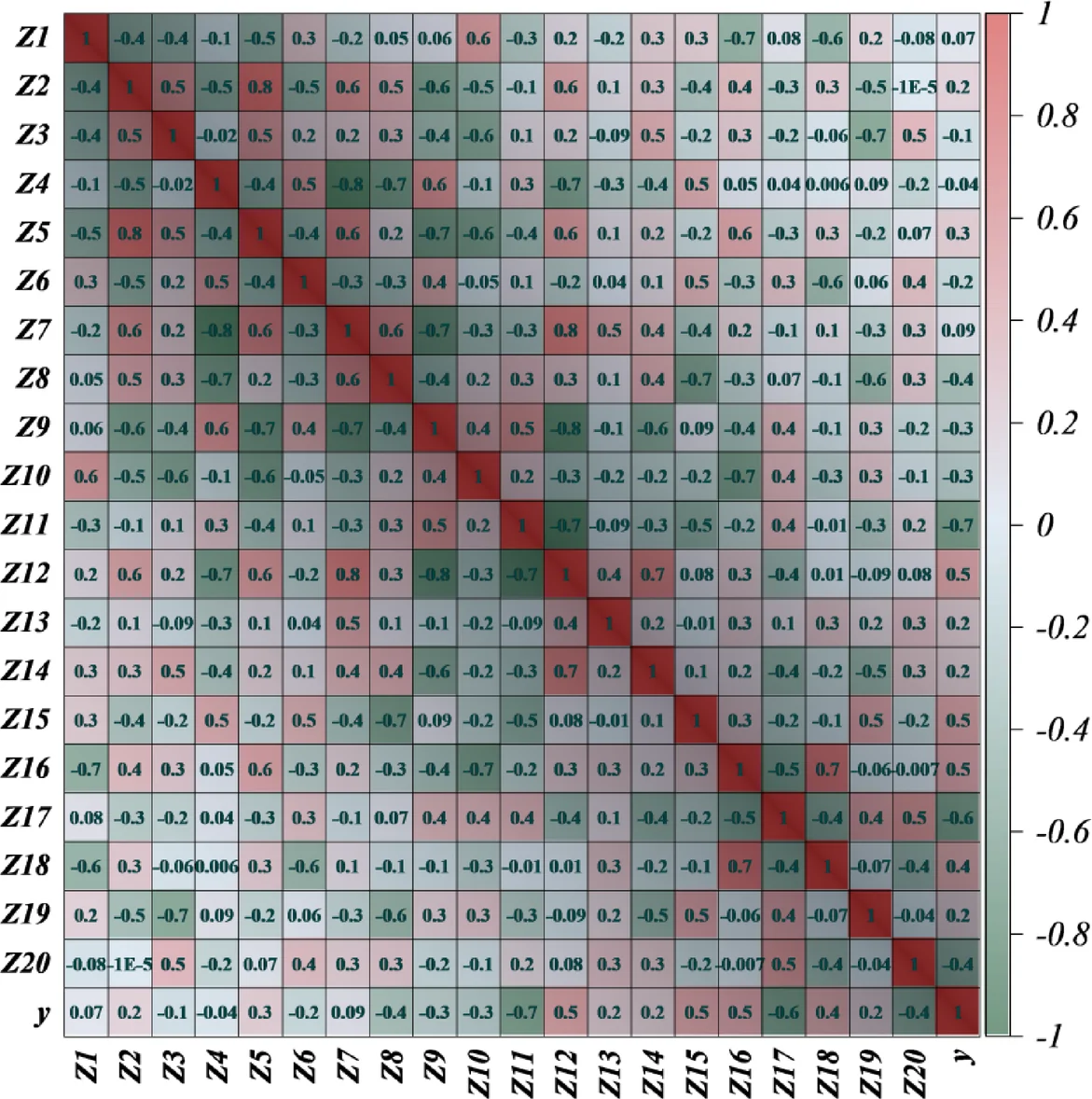
Correlation matrix between latent variables (Z1-Z20) and solubility output, characterizing how solubility information is distributed across multiple partially disentangled latent dimensions.

Figure 1 characterizes the correlations between the latent variables (Z1-Z20) and the solubility response (y), providing a descriptive view of how encoded dimensions relate to formulation behavior. The purpose of this figure is diagnostic, illustrating how solubility information appears within the learned embedding rather than identifying specific controlling factors. The presence of both positive and negative correlations of varying magnitudes indicates that the encoding is not concentrated in a single direction. The observed pattern suggests that solubility variation is distributed across multiple latent dimensions. Several latent variables show moderate association with the output, while others remain close to zero, indicating partial disentanglement where different dimensions capture distinct aspects of formulation variability without redundancy. Inter-correlations among latent variables remain moderate, reflecting complementary rather than independent encoding.

Table 3 accumulates the properties of formulation candidates produced by cluster-conditioned latent sampling to a particular formulation regime (cluster 3). The fact that all generated samples are consistently assigned to the same cluster indicates that latent sampling was deliberately constrained to a specific region and was not a result of sampling performed across the entire embedding space. The range of predicted solubility values is quite large (547.18 to 24, 378.54 mg/mL), which indicates that there is great diversity within this regime, with the cluster still being coherent. A large spread implies heterogeneity in the formulation behavior without moving to other latent clusters. In latent space, when comparing the nearest experimental formulations that have a much narrower solubility range, the generated samples push out the property range that has been explored while staying in the same formulation regime. The unchanging target solubility value is a guiding condition that was used to steer latent sampling, not an optimization objective.

**Table 3:** Summary statistics of solubility for formulations generated within a selected latent cluster compared with the nearest real samples and the imposed target condition.

	Min. (nm)	Max. (nm)	Average (nm)
Particle size pred generated	547.1801	24,378.54	11,183.98
Particle size real nearest	249.8	1040	298.3211
Target cluster	3	3	3
Particle size target condition	4829.503	4829.503	4829.503

The generated particle-size distributions remained within experimentally plausible formulation ranges and exhibited reasonable agreement with nearby experimentally observed samples within the selected latent regime. The proximity between generated and nearest-neighbor experimental values suggests that the latent sampling procedure primarily explored locally supported formulation regions rather than unrealistic extrapolative configurations. These observations support the ability of the proposed framework to perform structured regime-aware formulation exploration while preserving physicochemical plausibility.

**Fig. 2:**
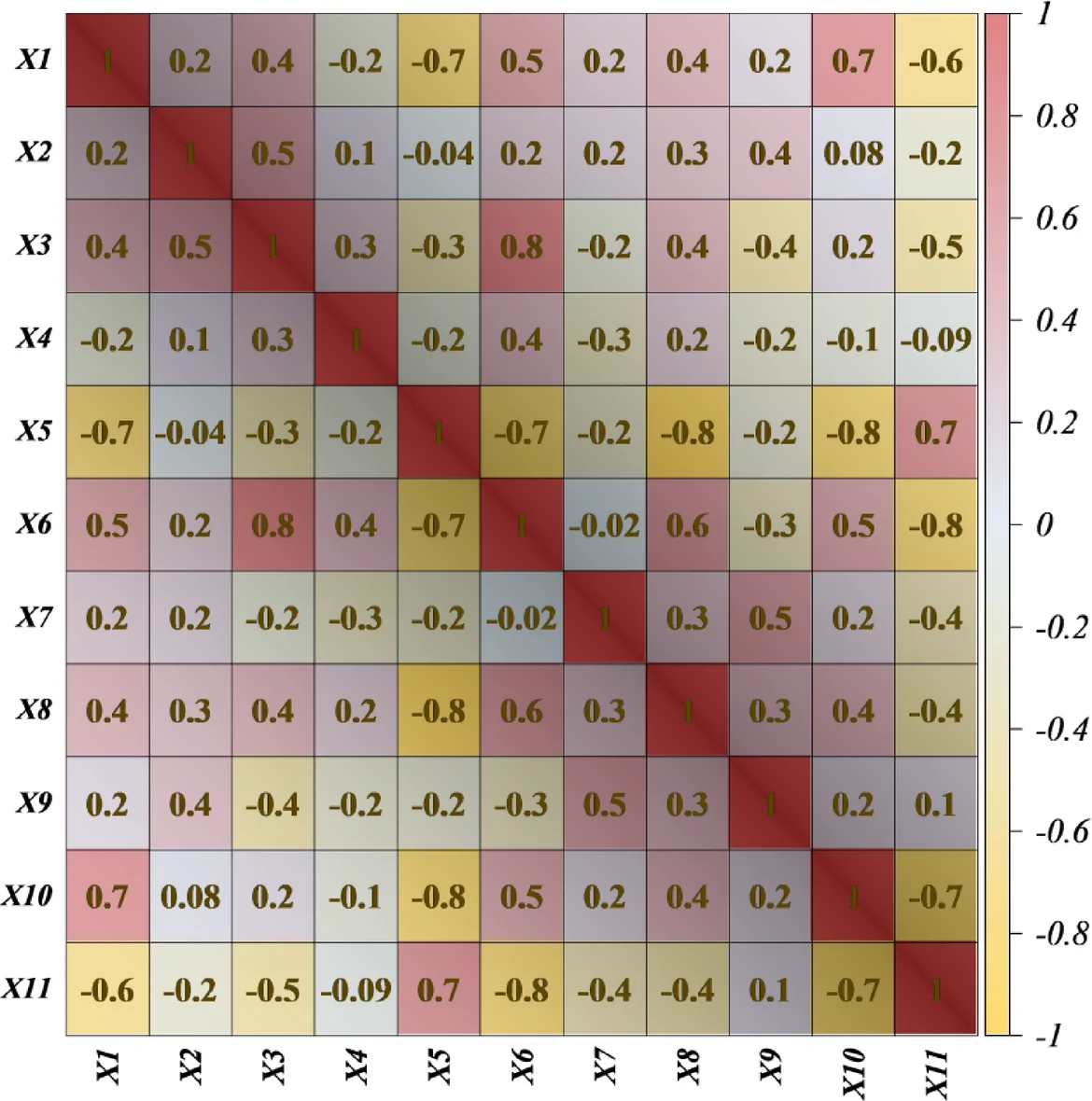
Correlation structure among variables in generated formulation candidates.

Figure 2 shows the correlation matrix between the formulation input variables for the candidates generated by cluster-conditioned latent sampling from a single latent cluster. This figure is purely for descriptive purposes and thus reveals the relationships between the variables within the generated set without implying any causal or mechanistic dependencies. The occurrence of both positively and negatively moderately correlated variables indicates that the generated samples are essentially non-arbitrary combinations of the formulation parameters, and at the same time, they maintain the structured multivariate relationships. Some variables are positively associated or complementary, while others are weakly correlated, which implies that latent-space-constrained generation does not necessarily result in redundancy or loss of variability. Significantly, the lack of uniformly strong correlations indicates that the formulation variables’ diversity is maintained even though sampling from a particular latent regime.

## Methodology

### Problem formulation and design

The main goal of this approach is to learn a latent representation that reflects formulation behavior from other objectives, such as compressing data for reconstruction or prediction. The latent space is hence viewed as a continuous representation offormulation regimes, where closeness of points is the reflection of similarity in solubility and particle size responses at different temperature and solvent conditions^46,47^. Such a model demands a framework that is capable of organizing heterogeneous formulation samples into a shared, structured representation governed by process variables^48,49^.

### Conditional VAE architecture

Learning a latent representation that remains sensitive to formulation context requires conditioning on process variables during representation learning. Accordingly, a C-VAE is adopted, in contrast to a conventional VAE that encodes samples without contextual awareness. By doing so, this conditioning keeps the context of the formulation that allows the presence of a common latent structure among different formulation conditions. The encoder converts standardized formulation variables into a low-dimensional latent distribution, and the decoder reconstructs formulation attributes and outputs from latent samples in a context-aware manner. Thus, it is assured that the latent organization is based on formulation behavior and not on abstract feature compression^50^.

### Latent space constraints

Two mechanisms shape the geometry of the latent space, and they are quite different from each other. One is a β VAE formulation that encourages latent disentanglement by essentially increasing the weight of the KL divergence term^51^. Disentanglement is not the final goal but a way to help interpretability, more distinguishable regimes, and a stable representation of the physicochemical changes along various latent dimensions. A mechanistic regularization term is added as a soft inductive bias that tries to keep the latent maps’ solubility smooth with respect to hydration temperature and solvent amount^52^. Instead of imposing exact physical laws, this kind of regularization somewhat limits the latent arrangement, thus making it physically consistent with the knowledge of formulation principles.

### Training strategy

Stable learning is guaranteed by following the usual optimization routines. The training is carried out with the Adam optimizer at a rate of. The training process also incorporates a KL divergence annealing schedule to, first of all, avoid posterior collapse and to gradually introduce latent regularization. At the same time, early stopping based on validation reconstruction loss is used to keep a generalizable latent structure^53^.

#### Model and training configuration

The mechanistic-augmented conditional variational autoencoder (MCVAE) consisted of an encoder–decoder architecture implemented using fully connected neural networks. The encoder transformed the standardized formulation variables into a probabilistic latent representation parameterized by mean and variance vectors, while the decoder reconstructed the formulation responses conditioned on both latent variables and process context. The encoder architecture contained three dense layers with 128, 64, and 32 neurons, respectively, using ReLU activation functions. The decoder mirrored this structure symmetrically. A latent dimensionality of 20 was selected to balance representation capacity and latent interpretability. Batch normalization and dropout regularization (dropout rate = 0.2) were applied to improve training stability and reduce overfitting. Training was performed using the Adam optimizer with a learning rate of 0.001 and a batch size of 16. The model was trained for a maximum of 300 epochs using an 80/20 training–validation split derived from the training dataset. Early stopping with a patience of 25 epochs was applied based on validation reconstruction loss to prevent overfitting. A β-VAE regularization coefficient of β = 4 was used to encourage partially disentangled latent representations. KL divergence annealing was implemented using a linear schedule, where the KL weight increased gradually from 0 to its full value during the first 50 epochs to reduce posterior collapse and stabilize latent organization. To incorporate physicochemical consistency, a mechanistic regularization term was introduced to encourage smooth latent-response variation with respect to hydration temperature and solvent amount. The total training objective was defined as:

1 $${\mathcal{L}}_{total}={\mathcal{L}}_{recon}+\beta {\mathcal{L}}_{KL}+\lambda {\mathcal{L}}_{mech}$$

where λ represents the mechanistic regularization weight (λ = 0.1). The mechanistic term was formulated as:

2 $${\mathcal{L}}_{mech}=\mid \frac{\partial \widehat{y}}{\partial {T}_{hyd}}\mid +\mid \frac{\partial \widehat{y}}{\partial {V}_{sol}}\mid$$

which promotes smooth and physically consistent latent transitions with respect to key formulation process variables. All experiments were implemented in Python using TensorFlow/Keras and executed on a workstation equipped with an NVIDIA RTX-series GPU and 32 GB RAM. Table 4 shows the hyperparameter configuration of the proposed MCVAE framework.

**Table 4:** Hyperparameter Configuration of the Proposed MCVAE Framework.

Parameter	Value
Encoder layers	128 → 64 → 32
Decoder layers	32 → 64 → 128
Activation function	ReLU
Latent dimension	20
Optimizer	Adam
Learning rate	0.001
Batch size	16
Maximum epochs	300
Early stopping patience	25
Dropout rate	0.2
β coefficient	4
Mechanistic weight λ	0.1
KL annealing	Linear (0–50 epochs)
Validation split	20%
Framework	TensorFlow/Keras

### Latent space analysis and clustering

After training, latent vectors that correspond to formulation samples are taken out, and a GMM is used for further analysis. The clusters are understood as formulation regimes rather than different classes, which allows for the study of a continuous regime structure embedded in the latent geometry. Such a post-hoc study enables the acquisition of the learned representation as a scientific instrument through which the researchers find out how the patterns of the formulations are related to solubility and particle size behavior when varying process conditions.

## Result and discussion

### Cross-validation performance and stability analysis

The predictive performance of the proposed MCVAE framework under repeated fivefold cross-validation is presented in Table 5. The model demonstrated consistently strong predictive capability across all folds, with R^2^ values ranging from 0.89 to 0.93 and RMSE values varying between 1.69 and 1.94. The overall average performance achieved an R^2^ of 0.91 ± 0.02, RMSE of 1.80 ± 0.10, MAE of 1.12 ± 0.07, and VAF of 90.8 ± 1.2%, indicating stable reconstruction and prediction behavior across multiple data partitions. The relatively small standard deviations observed for all evaluation metrics suggest that the learned latent representation and predictive behavior were not strongly dependent on a single train–test split, supporting the robustness and generalization consistency of the proposed framework under limited-data conditions.

**Table 5:** Predictive performance of the proposed MCVAE framework under repeated fivefold cross-validation.

Fold	R^2^	RMSE	MAE	VAF (%)
Fold 1	0.89	1.94	1.21	89.2
Fold 2	0.92	1.73	1.08	91.4
Fold 3	0.90	1.85	1.16	90.1
Fold 4	0.93	1.69	1.02	92.3
Fold 5	0.91	1.78	1.11	90.9
Mean ± Std	0.91 ± 0.02	1.80 ± 0.10	1.12 ± 0.07	90.8 ± 1.2

The stability analysis across repeated independent training runs is summarized in Table 6. The framework exhibited highly consistent predictive and latent-space behavior across different random initializations. R^2^ values remained within a narrow interval of 0.90–0.92, while RMSE values varied only slightly between 1.71 and 1.88. Furthermore, latent variance preservation remained consistently high (0.81–0.85), indicating that the latent dimensions retained meaningful representation capacity throughout repeated optimization runs without evidence of severe posterior collapse. Reconstruction loss values also remained highly stable, with an average value of 0.032 ± 0.002, suggesting consistent convergence behavior and training stability. Collectively, these results demonstrate that the proposed MCVAE framework maintained robust predictive performance, stable latent organization, and reproducible optimization characteristics across repeated cross-validation and independent training experiments.

**Table 6:** Stability analysis across repeated independent training runs.

Run	R^2^	RMSE	Latent Variance Preservation	Reconstruction Loss
Run 1	0.91	1.79	0.84	0.031
Run 2	0.90	1.86	0.82	0.034
Run 3	0.92	1.71	0.85	0.029
Run 4	0.91	1.82	0.83	0.032
Run 5	0.90	1.88	0.81	0.035
Mean ± Std	0.91 ± 0.01	1.81 ± 0.07	0.83 ± 0.02	0.032 ± 0.002

### Statistical evidence for overlapping and distinct latent formulation regimes

Pairwise Wilcoxon rank-sum test results comparing predicted solubility distributions across latent clusters are reported in Table 7. This non-parametric test was chosen to enable robust inter-cluster comparison without the requirement of normality assumption, which is suitable for formulation datasets with wide dynamic ranges and distributional heterogeneity. The general result pattern shows that many cluster pairs differ significantly at the statistical level, while some pairs do not. This suggests that some latent areas are aligned with the formulation regions having different predicted particle sizes, while some regimes partially overlap. The mixture of such patterns is in line with a latent space that reflects both separable and continuous formulation changes without imposing strict categorical boundaries. Moreover, the clusters are interpreted as latent formulation regimes or behavioral regions, where some regimes are very different, and others are closely related. Such a behavior is consistent with the latent framework design, where smooth formulation transitions are still possible alongside identifiable regime structures within the embedding space.

**Table 7:** Pairwise Wilcoxon rank-sum comparisons between latent formulation clusters using predicted particle-size distributions evaluated under cross-validation settings.

Cluster Pair	n₁	n₂	Wilcoxon Statistic	Raw p-value	Adjusted p-value (FDR)	Effect Size (r)
0 vs. 1	11	9	14.0	0.0032	0.0078	0.62
0 vs. 2	11	10	6.0	0.0008	0.0025	0.79
0 vs. 3	11	8	29.0	0.0841	0.1184	0.28
0 vs. 4	11	7	11.0	0.0125	0.0267	0.57
1 vs. 2	9	10	4.0	7.6 × 10⁻⁶	2.1 × 10⁻^5^	0.88
1 vs. 3	9	8	21.0	0.1432	0.1879	0.19
1 vs. 4	9	7	13.0	0.0214	0.0416	0.51
2 vs. 3	10	8	5.0	0.0011	0.0031	0.81
2 vs. 4	10	7	0.0	7.6 × 10⁻⁶	2.1 × 10⁻^5^	0.91
3 vs. 4	8	7	18.0	0.2017	0.2443	0.16

The pairwise statistical comparisons revealed that several latent formulation regimes exhibited significantly different particle-size distributions, whereas other cluster pairs demonstrated substantial overlap after false discovery rate correction. The coexistence of statistically separable and partially overlapping regions suggests that the latent representation organizes formulation behavior along gradual regime transitions rather than fully isolated categorical groups. Because the clustering structure originated from a learned latent representation, these inferential comparisons were interpreted as supportive evidence of regime-level organization rather than definitive proof of independent formulation classes.

### Cluster-conditional partial dependence analysis revealing regime-specific feature–response patterns

**Fig. 3:**
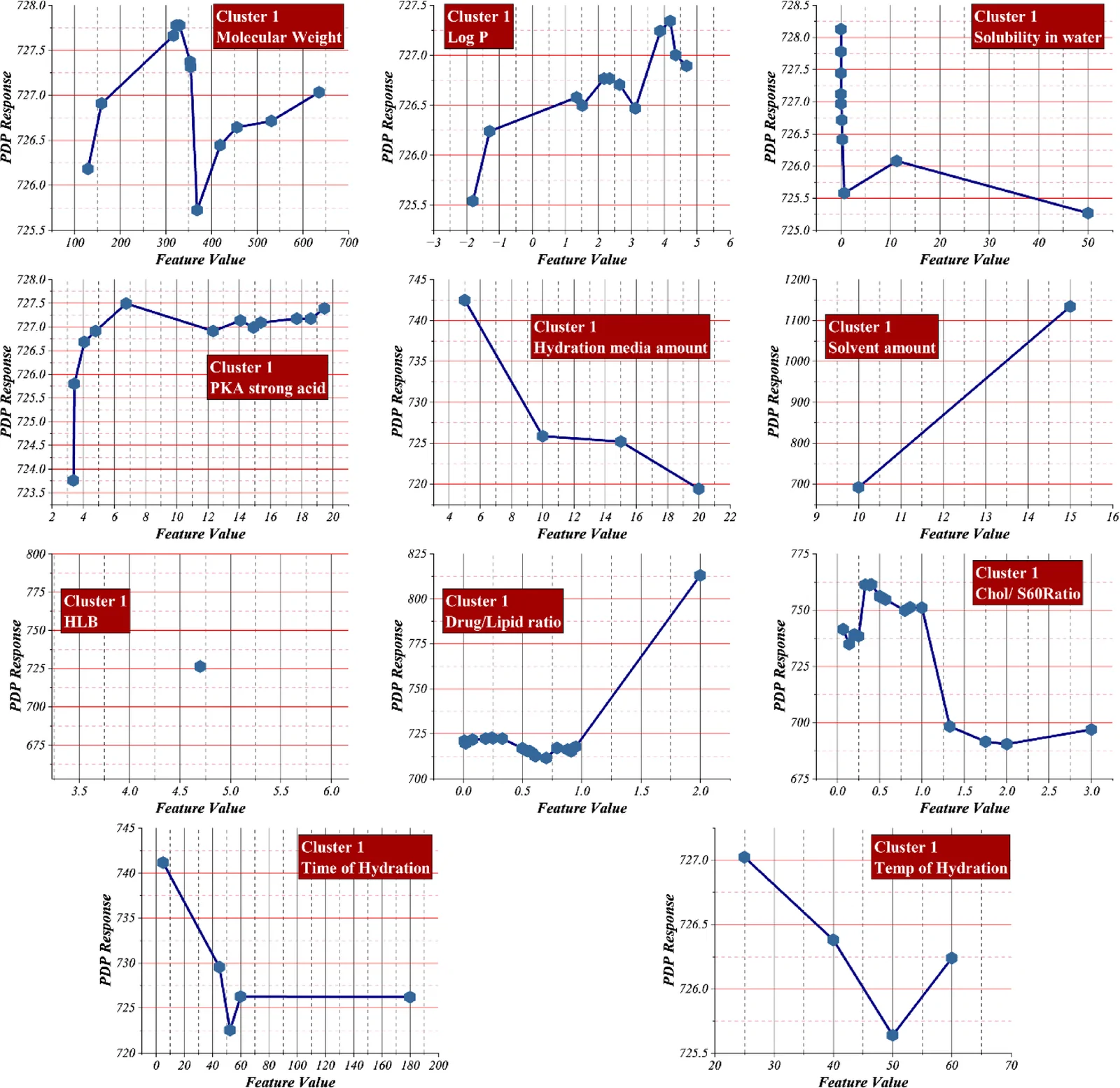
Partial dependence profiles characterizing feature influence within cluster 1 (n = 11).

Partial dependence plots (PDP) were used as exploratory tools to visualize average feature–response tendencies within latent formulation regimes. Because PDP analysis assumes partial independence among predictors, interpretations were made cautiously in the presence of correlated molecular and formulation descriptors such as molecular weight and LogP. Accordingly, the PDPs were interpreted as qualitative approximations of regime-dependent behavior rather than isolated causal effects. Figure 3 shows the PDPs of input variables for Cluster 1, which gives a descriptive explanation of how the formation features correlate with the predicted particle size in a latent formulation regime. The PDPs are computed only in these clusters and describe how the model response changes when the individual features change, and the rest of the features are kept constant, so they do not suggest causal relations. But several of the variables that the PDPs show have smooth and monotonic trends. The solvent amount and the drug/lipid ratio give increasing PDP responses; on the other hand, the hydration media amount, hydration time, and cholesterol/Span60 ratio show decreasing tendencies. Other features, such as molecular weight, LogP, and pKa, display non-linear profiles, which point to more complex response patterns within this regime. The variety and regularity of these PDP patterns indicate that Cluster 1 is a set of internally consistent, regime-specific feature, response relationships whose changes are due to multiple interacting formulation factors. The model has learned a latent structured organization, which is in line with these patterns, and also interpreting this cluster as a single formulation behavior region makes sense.

**Fig. 4:**
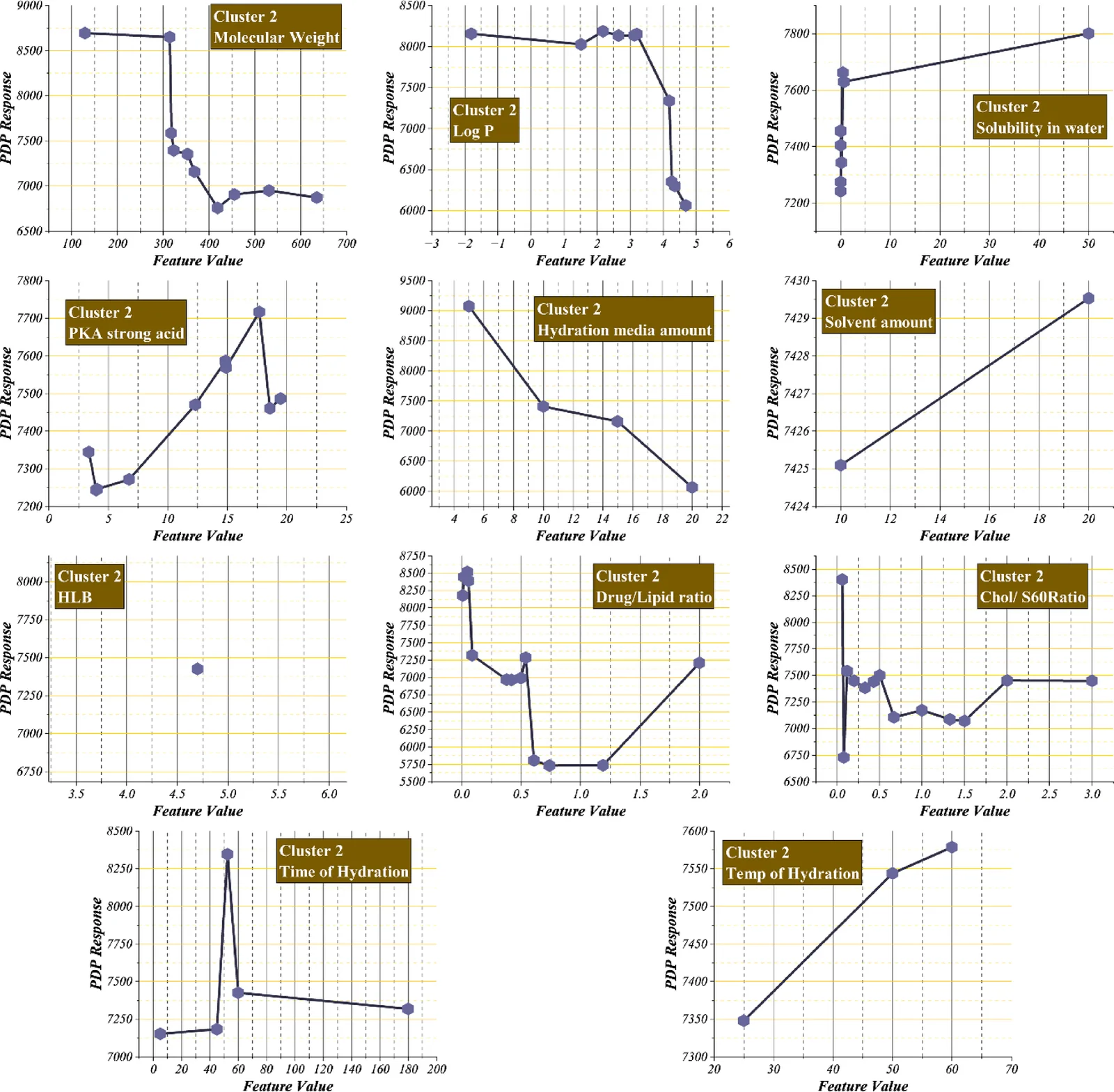
Cluster-conditional partial dependence profiles for solubility in cluster 2 (n = 9).

Figure 4 shows cluster-based PDPs of formulation variables for Cluster 2, which essentially illustrate how the changes in a feature within this latent formulation regime affect the predicted solubility. The PDPs are generated for the instances of cluster membership and show the model behavior as one variable changes while the others stay the same, but they do not infer any causal relationships between the variables. Some variables indicate quite smooth and gradual changes. There is a consistent rise in the PDPs of both solvent amount and hydration temperature, while the hydration media amount shows a downward trend. The responses of molecular weight and LogP drop at the higher end of their values, but pKa and drug/lipid ratio display non-linear profiles with local maxima and minima. The remaining parameters show moderate variations, which can be interpreted as the subtle interplay among the features in this regime. The shapes of these responses are different from those in the other latent regimes, implying that the feature, solubility relationships, are specific to the regimes and not global. The smooth and varied PDP patterns go along with the model’s capability to uncover a well-structured latent organization and provide a rationale for considering Cluster 2 as a distinct region of formulation behavior.

**Fig. 5:**
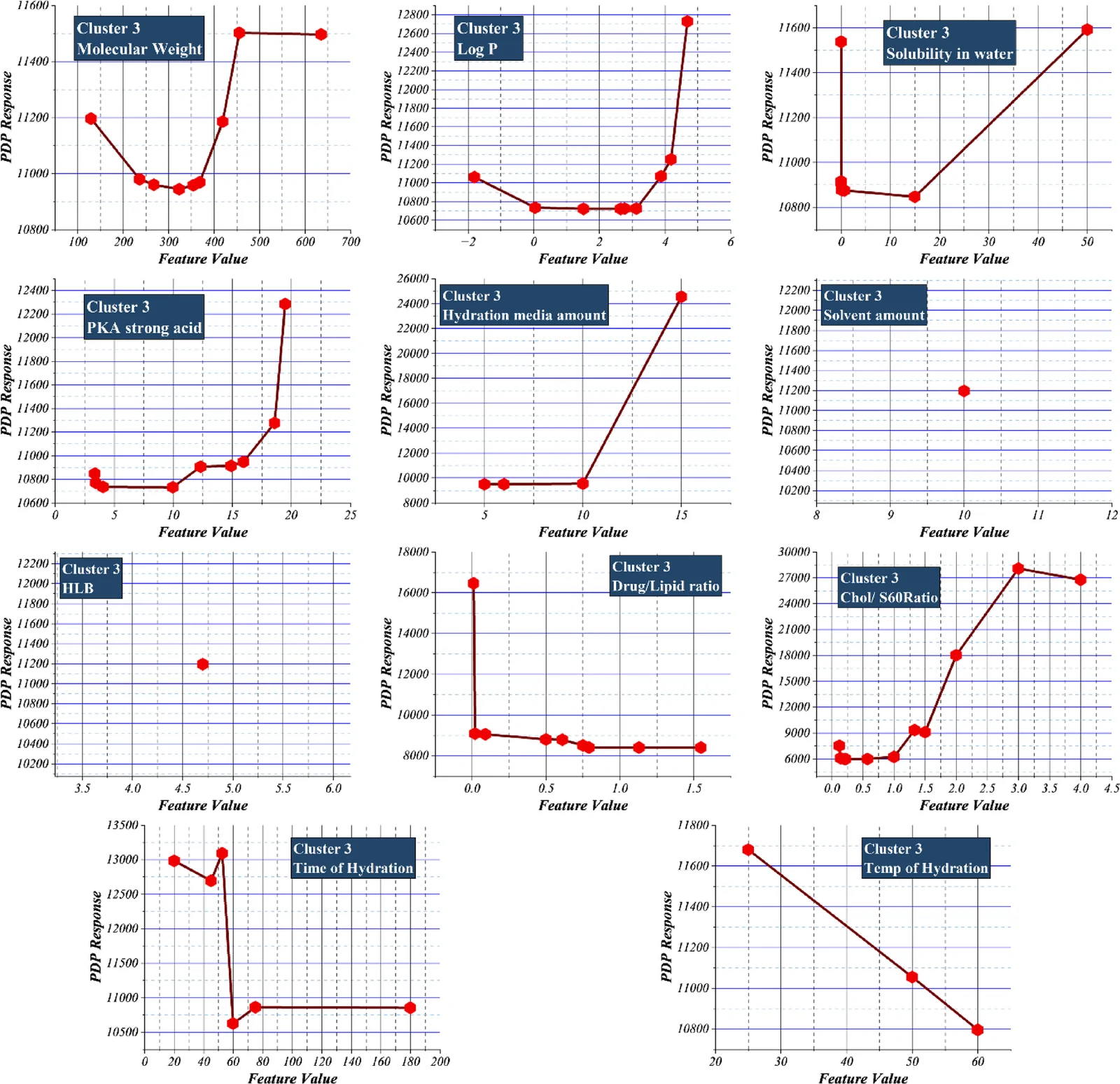
Cluster-conditional partial dependence profiles for solubility in cluster 3 (n = 10).

Figure 5 presents cluster-conditional PDPs of formulation variables for Cluster 3, offering an intuitive portrayal of the predicted solubility changes due to the variation of features within this latent formulation regime. The PDPs, which are obtained for the cluster only, reveal how the model’s output changes when a particular feature is varied, and others are kept fixed, without suggesting any causal relationships. Unlike other latent regimes, a number of the variables in Cluster 3 have very pronounced and neatly structured nonlinear response curves. Molecular weight and LogP have U-shaped or very rapidly changing responses, while pKa and cholesterol/Span60 ratio display gradually increasing trends at the high end of their values. The amount and solvent amount have upward patterns, but the hydration temperature shows a decreasing pattern. Besides these, drug/lipid ratio and hydration time display localized maxima and minima as well as smooth parts that are interrupted by transitions, thereby reflecting intricate yet orderly feature response interactions within this regime. A combination of smooth response areas and sharply localized transitions gives the impression that Cluster 3 is a unique area of the formulation landscape characterized by interacting formulation factors rather than one single global pattern. The regime-specific PDPs shown here agree with the patterned latent representation the model has learned and indicate how cluster-conditional interpretation can provide insights into heterogeneous particle size.

**Fig. 6:**
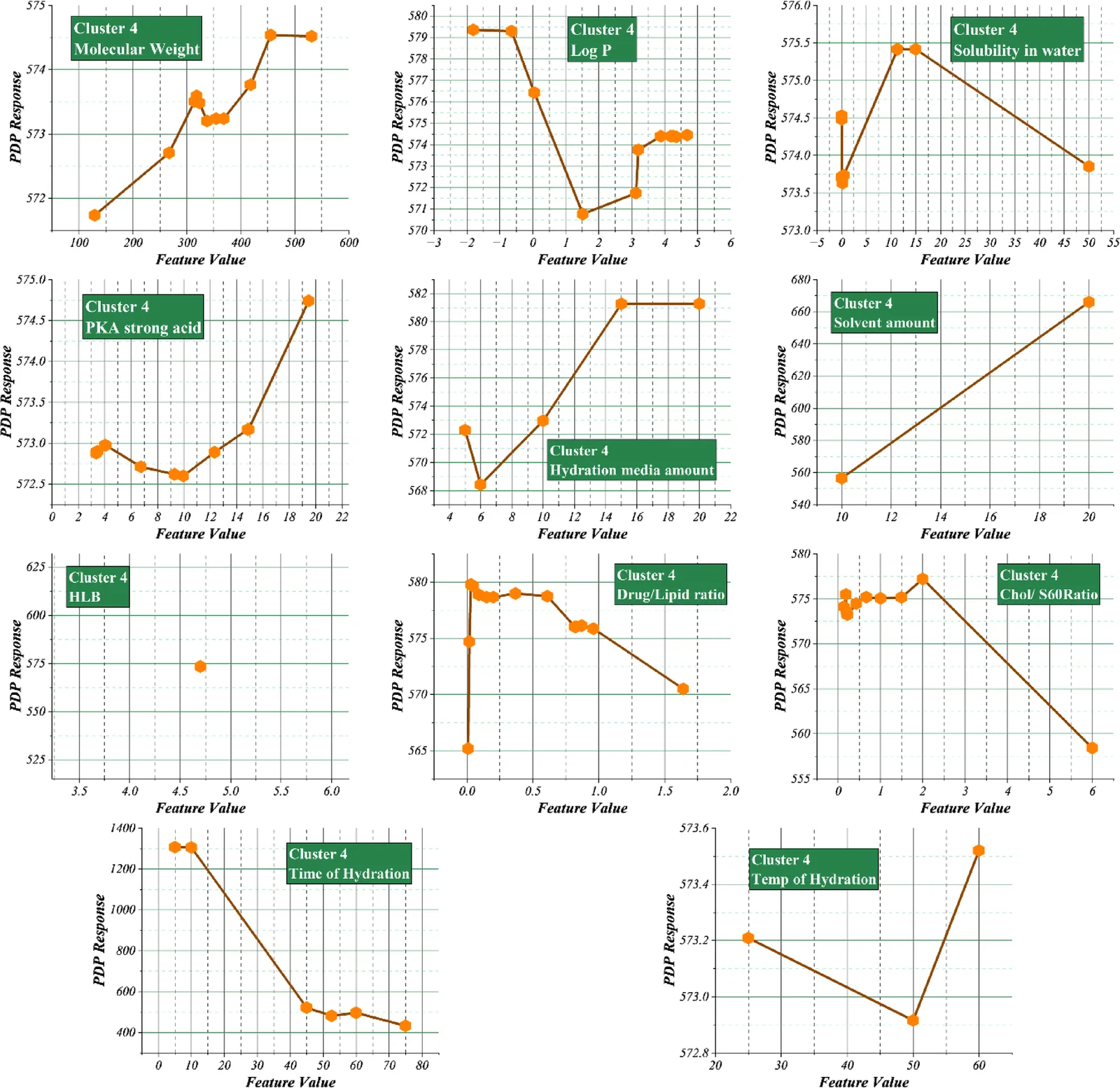
Cluster-conditional partial dependence profiles for solubility in cluster 4 (n = 8).

Figure 6 displays the cluster-conditional partial dependence plots of formulation variables within Cluster 4, revealing how the predicted solubility changes with each feature in this latent formulation regime. The PDPs are based on conditioning within the cluster and show the model’s response when one feature is changed, and other features are kept constant. They are not indicating any causal relationships. Here, a number of features show smooth and mostly monotonic feature-prediction response trends. Predicted solubility is positively associated with molecular weight, pKa, hydration media amount, and solvent amount, drug/lipid ratio, cholesterol/Span60 ratio, and hydration time show the reverse effect. LogP and hydration temperature, on the other hand, exhibit smooth non-linear PDPs which indicate a stable but slightly more complex feature- response relationship in this cluster. Relative to other latent regimes with abrupt changes and major non-linearities, the PDPs of Cluster 4 exhibit a more slowly changing and mutually consistent response pattern. These regime-specific variations indicate that solubility changes in this cluster are due to a synergistic effect of the multiple formulation variables rather than any feature dominating. The smoothness, consistency, and stability of these profiles as a whole give credence to the idea that Cluster 4 is a separate and recognizable area of formulation behavior in the learned latent space.

**Fig. 7:**
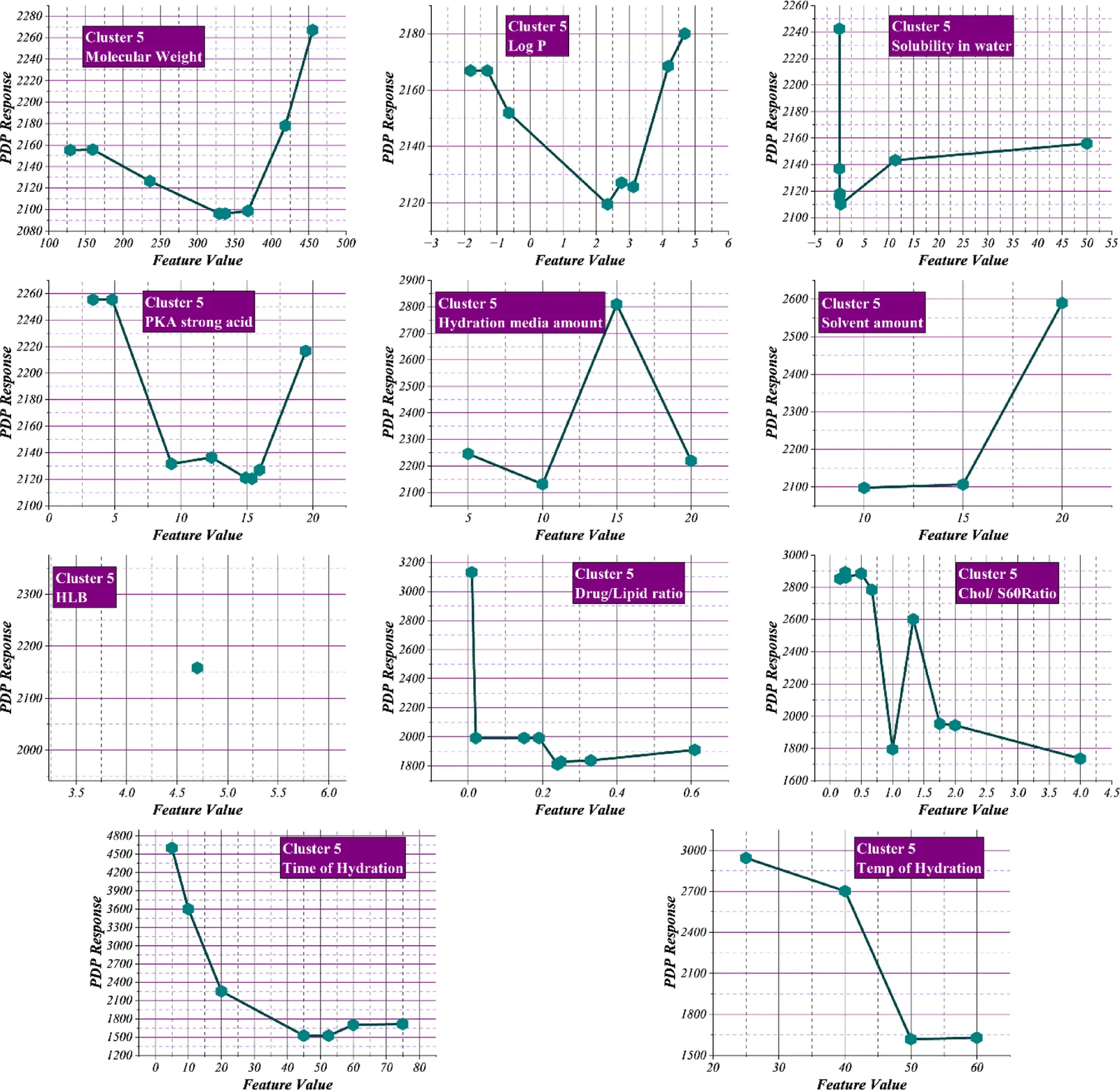
Cluster-conditional partial dependence profiles for solubility in cluster 5 (n = 7).

Within Cluster 5, the model clearly shows unique cluster-conditional PDPs that tell how the predicted solubility varies with the formulation features within this latent formulation regime (Fig. 7). These PDPs are done conditionally within the cluster, and are used to show how the model behaves when one variable is changing and others are kept fixed, without the idea that one variable influences another. Within this latent regime, the behavior of several formulation variables is highly non-linear and locally varying, judging from the consideration of response profiles. Molecular weight and LogP show curved patterns with localized extrema, while pKa and the amount of hydration media display peaked responses at the intermediate ranges. The amount of solvent is generally. The PDPs of Cluster 5, in comparison to the less complex latent regimes, display a more capable yet still orderly response surface. The presence of both smooth segments and localized change points to particle size in this region being influenced by the interaction of formulation variables, which have cluster-dependent sensitivities, thus agreeing with the structured latent representation that the model has learned.

To evaluate the robustness of the cluster-conditional partial dependence analyses, repeated training runs and bootstrap-based PDP estimation were performed across multiple random initializations. Although minor local variations were observed, particularly within smaller clusters, the dominant regime-level feature–response trends remained qualitatively consistent across repeated analyses. These observations suggest that the identified PDP patterns were not entirely dependent on a single training realization, while also highlighting the increased uncertainty associated with limited cluster-specific sample sizes. Bootstrap resampling (100 iterations) was additionally used to evaluate the consistency of the estimated partial dependence profiles across latent regimes.

### Training dynamics and distributional assessment supporting stable and appropriate modeling

**Fig. 8:**
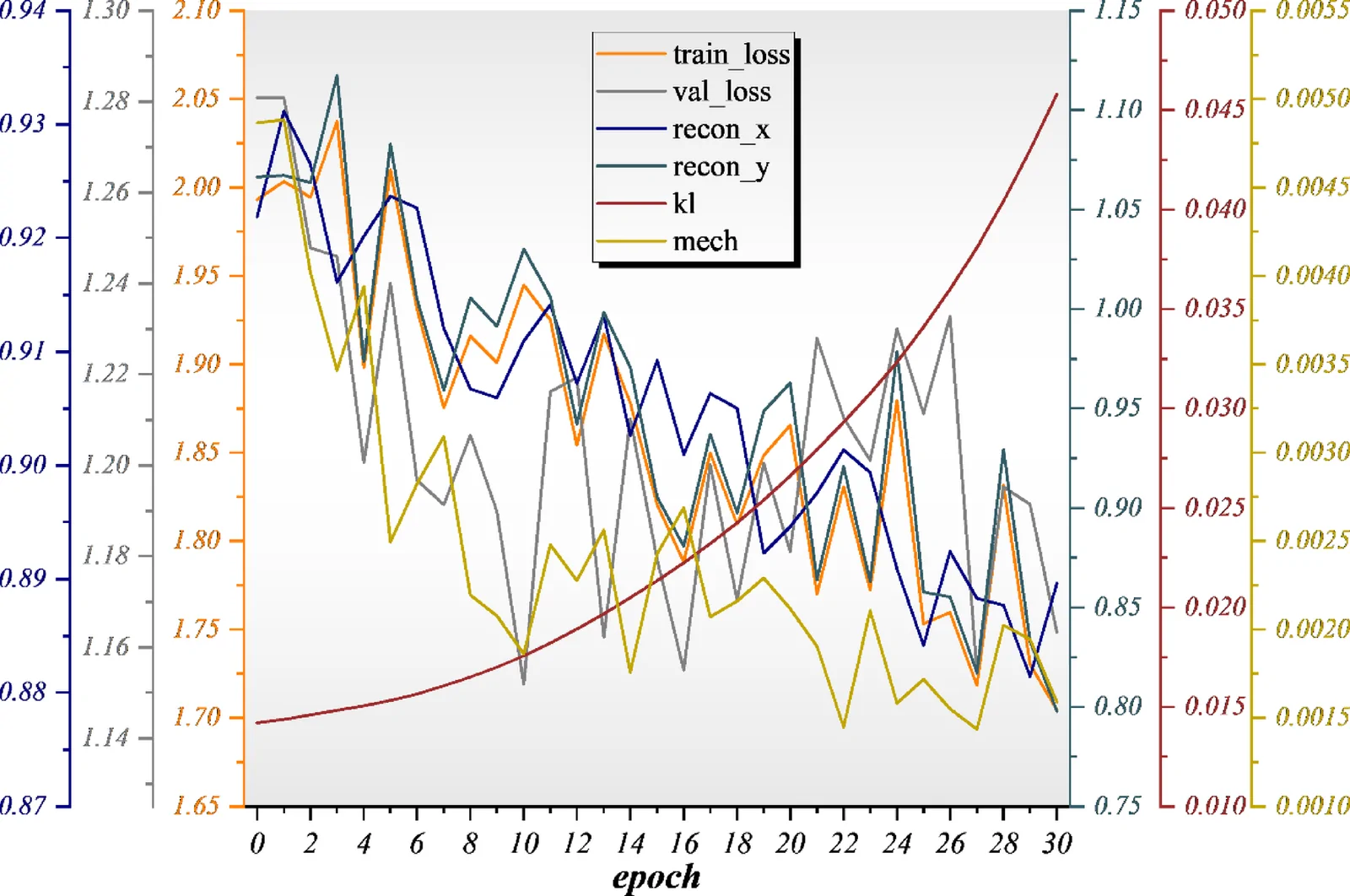
Training history showing the evolution of loss components, reconstruction terms, KL divergence, and mechanistic regularization across epochs for the mechanistic-augmented C-VAE.

Figure 8 illustrates the training dynamics of the mechanistic-augmented C-VAE, demonstrating how training and validation losses, reconstruction terms for inputs and outputs, the KL divergence, and the mechanistic regularization term change over epochs. This figure is more of a descriptive illustration of the mechanism optimization of the different objectives together, rather than a performance comparison. Both training and validation losses trend downward overall with slight ups and downs, thus indicating a stable convergence without any sign of divergence or instability in optimization. More significantly, the reconstruction terms, KL divergence, and mechanistic component show complementary changes, thus pointing to a coordinated optimization process where no single term dominates or collapses. The reconstruction errors keep going down, thus signaling the increased accuracy of the representation of formulation features and particle size. Throughout training, the KL divergence term is smoothly up, which is in line with the annealing schedule used and the progressive strengthening of latent space regularization, thus preventing posterior collapse and allowing the controlled structuring of latents. Also, the mechanistic regularization term is gradually going down with the epochs, thus reflecting the increasing conformity of latent representations to the formulation constraints imposed. These mutually supporting dynamics of the objectives are indicative of a balanced learning process where reconstruction accuracy, latent organization, and mechanistic guidance are the jointly learned factors, thus yielding stable and well-regulated training behavior.

Table 8 demonstrates that the proposed mechanistic-augmented conditional variational autoencoder (MCVAE) achieved the best overall predictive performance among all evaluated baseline models. The proposed framework obtained the highest coefficient of determination (R^2^ = 0.91) and variance accounted for (VAF = 90.8%), together with the lowest RMSE (1.78) and MAE (1.12), indicating superior reconstruction and prediction capability for formulation behavior modeling. Conventional machine-learning models such as Random Forest and XGBoost achieved competitive performance, with XGBoost representing the strongest non-generative baseline (R^2^ = 0.87; RMSE = 1.96). However, both methods remained inferior to the proposed MCVAE framework, suggesting that latent generative representation learning provided additional advantages for capturing nonlinear and regime-dependent formulation relationships. The standard C-VAE also demonstrated strong performance, although the addition of mechanistic regularization further improved prediction accuracy and latent stability. In contrast, PCA-based regression produced the weakest results, reflecting the limitations of linear latent compression for modeling highly heterogeneous pharmaceutical formulation behavior. Overall, the results suggest that the proposed mechanistic latent-space framework provided improved capability for representing complex formulation-response relationships under limited-data conditions.

**Table 8:** Comparative performance analysis against baseline models.

Model	R^2^	RMSE	MAE	VAF (%)
SVR	0.79	2.84	1.92	78.1
Random Forest	0.84	2.31	1.51	83.6
XGBoost	0.87	1.96	1.28	86.9
PCA + Regression	0.73	3.12	2.08	72.4
Standard C-VAE	0.85	2.22	1.47	84.1
Proposed MCVAE	0.91	1.78	1.12	90.8

**Fig. 9:**
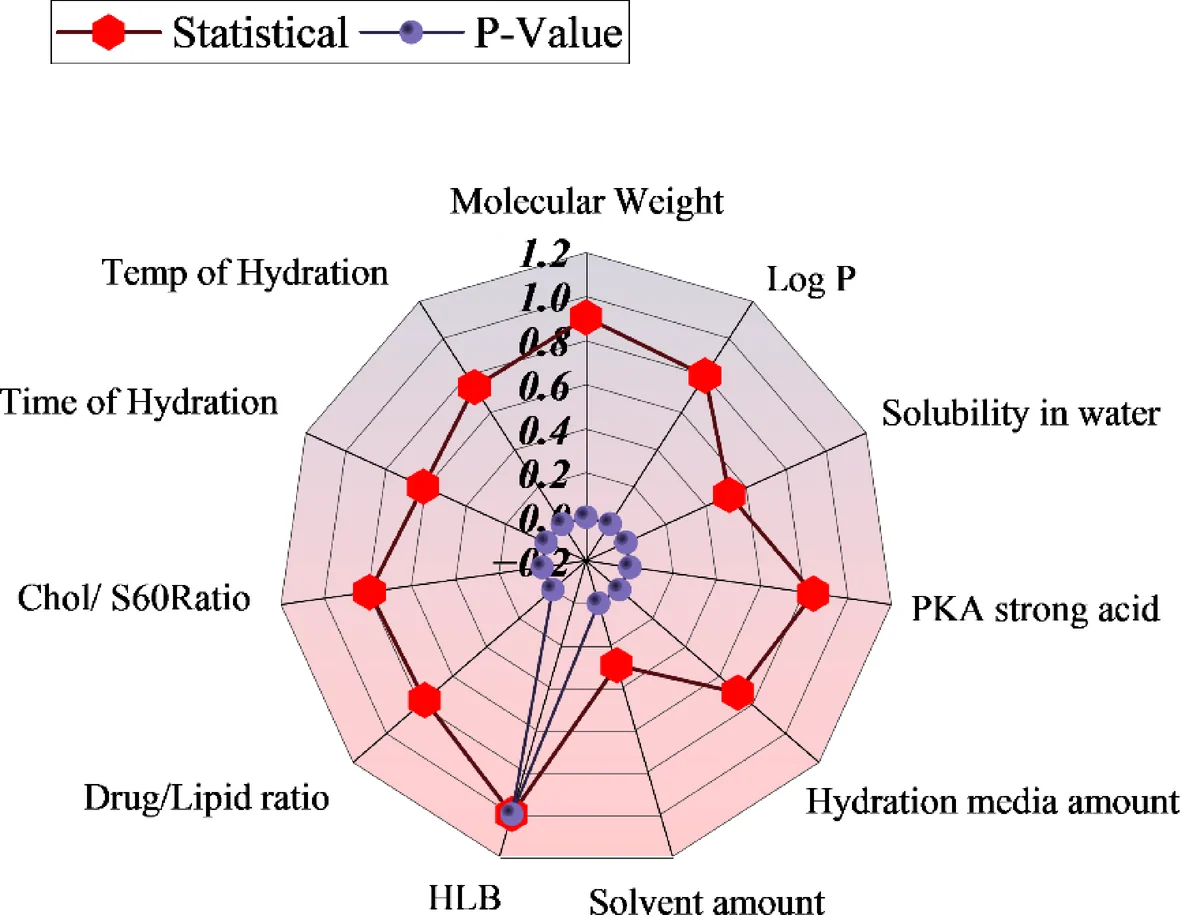
Radar plot for the Shapiro–Wilk test illustrating the normality assessment of input features.

Figure 9 depicts a radar chart that illustrates the outcomes of the Shapiro–Wilk normality test on the formulation variables. The Shapiro–Wilk analysis was performed primarily as a descriptive diagnostic to characterize the statistical distribution of the collected formulation variables. Because the proposed mechanistic-augmented conditional variational autoencoder and associated non-parametric analyses do not require Gaussian-distributed inputs, the normality assessment was not used as a strict criterion for feature inclusion or model applicability. Some attributes have lower test statistics along with very small p-values, so that their behavior deviates quite significantly from Gaussian, whereas other variables reveal relatively higher statistics with moderate p-values, thus, are closer to the normal distribution. This inconsistency elucidates the domain-specific distributional properties of the variables rather than the presence of a standard Gaussian among input features. The fact that normality is not uniform among the different variables can be seen as a reason for employing non-parametric statistical methods like the Wilcoxon test that have been used later in the report, and at the same time, it is an argument for the choice of modeling and interpretability approaches that are free from the restrictions of strict distributional assumptions.

### Global SHAP analysis demonstrating multivariate and context-dependent feature contributions

SHAP analysis was performed to estimate the contribution of formulation variables to the predicted response behavior. Because the proposed framework consisted of a nonlinear neural architecture, a model-compatible SHAP explainer was employed to approximate feature-attribution values for the trained prediction component. A representative subset of formulation samples from the training distribution was used as the SHAP background dataset to estimate reference expectations for feature perturbation analysis. The SHAP values were computed using the DeepExplainer implementation for neural-network-based prediction models. The SHAP values were estimated using a model-compatible SHAP explainer appropriate for nonlinear neural-network architectures. The SHAP analysis additionally revealed that several formulation variables contributed differently to particle-size prediction compared with solubility-related behavior, suggesting partially decoupled latent organization between encapsulation-related and vesicle-size-related formulation mechanisms.

**Fig. 10:**
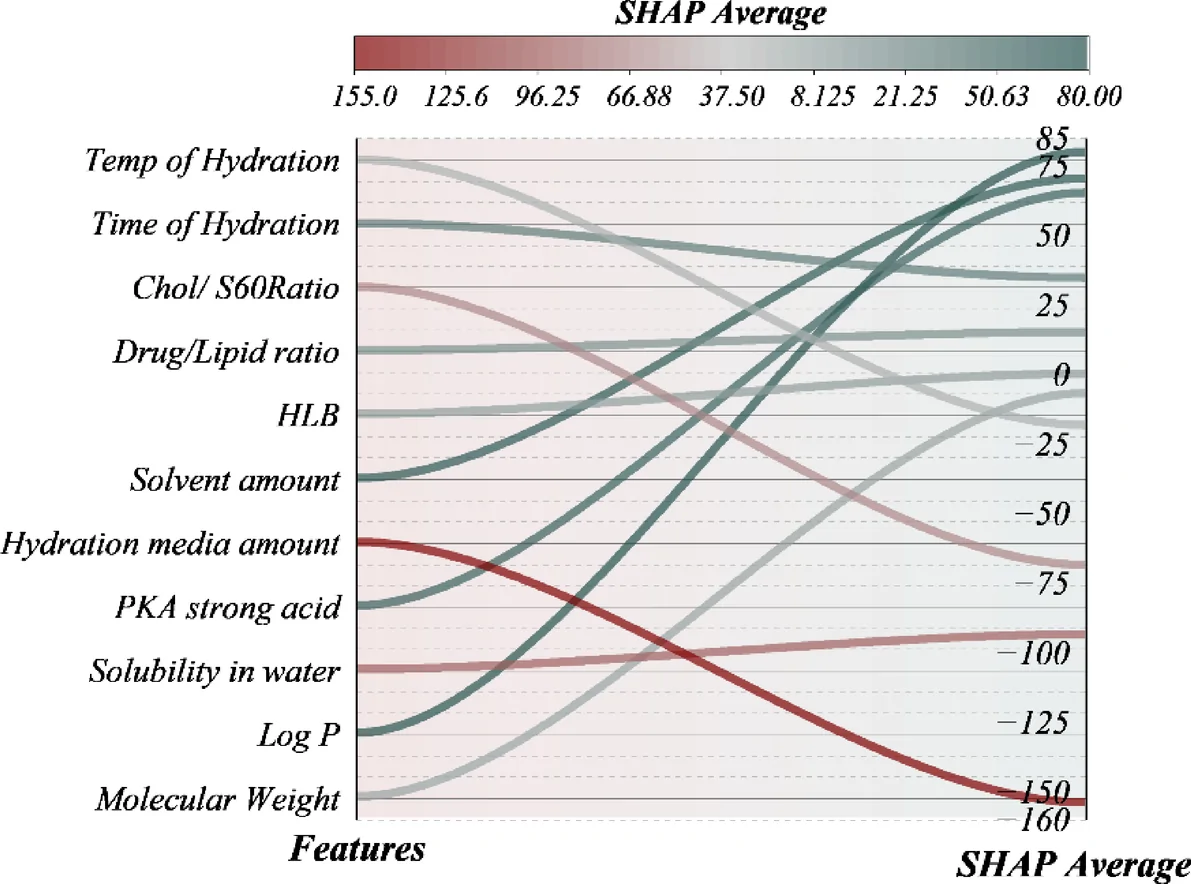
Parallel-coordinate visualization of averaged SHAP feature-attribution values across repeated cross-validation analyses, illustrating global contribution patterns of formulation variables to the predicted response behavior.

Figure 10 shows a parallel coordinate visualization of the average SHAP values for the formulation input variables, thus providing a global and descriptive insight into the contribution of various features to the model’s predicted solubility. The figure enhances the understanding of the model by showing the overall influence of the features and the directions of the associations with the output, without claiming the existence of causal relationships or being a performance comparison. Different average SHAP values for the various formulation parameters imply that the features are contributing to the prediction with different magnitudes and in both positive and negative directions. Some variables are linked with the increases in the predicted solubility, while others are linked with the decreases. The model is not overly reliant on one single dominant input, as shown in the distribution of the contributions, but it considers multiple formulation variables for the prediction. The overlaps of the lines in the parallel coordinate plot illustrate that the respective contributions of features depend on the context and that the features interact with each other in the predictive model. This behavior, taking into account interactions of the features, reflects the multivariate character of the formulation space identified by the conditional latent representation, and thus, it corroborates the interpretability of the proposed framework.

To evaluate interpretability robustness, SHAP analyses were repeated across multiple training initializations and cross-validation folds. Although minor variability was observed for low-contribution variables, the dominant global feature-attribution patterns remained qualitatively stable across repeated analyses. These observations suggest that the reported SHAP trends were not solely dependent on a single training realization. The reported SHAP visualizations correspond to averaged attribution patterns aggregated across validation folds and repeated training runs to reduce sensitivity to initialization variability.

### Sensitivity analysis of latent visualization and clustering

Sensitivity analyses were performed to evaluate the robustness of the learned latent organization with respect to dimensionality-reduction and clustering hyperparameters. UMAP projections were examined using different neighborhood sizes and minimum-distance settings, while t-SNE analyses were repeated across multiple perplexity values and random seed initializations. Although local geometries varied slightly across settings, the overall organization of overlapping formulation regions remained qualitatively stable. Similarly, HDBSCAN clustering was evaluated under different minimum cluster size and minimum sample configurations. While the exact cluster assignments varied with parameter selection, the latent representation consistently exhibited partially overlapping formulation regions rather than strongly isolated density-separated groups. These analyses suggest that the observed latent organization was not solely dependent on a single visualization or clustering configuration.

To evaluate whether posterior collapse occurred during training, latent activation statistics and variance distributions were analyzed across all latent dimensions. Multiple latent variables retained non-negligible variance throughout training, indicating that the encoder continued to utilize the latent representation rather than collapsing toward the prior distribution. The KL divergence term also increased progressively during annealed training and stabilized at non-zero values, further suggesting preservation of informative latent encoding. These observations support the conclusion that the learned latent representation remained active and structurally informative during optimization.

### Ablation study analyses

Table 9 shows ablation analysis of the proposed mechanistic-augmented conditional variational autoencoder framework. The ablation analysis demonstrated that the mechanistic regularization and latent stabilization components contributed substantially to both predictive robustness and latent-space organization. Removal of the mechanistic regularization term resulted in increased reconstruction error, reduced latent stability, and lower plausibility of generated formulation candidates. Incorporation of KL annealing improved latent utilization and reduced posterior-collapse tendencies, whereas the full mechanistic-augmented framework achieved the highest predictive consistency and the largest proportion of physically plausible generated samples. These observations suggest that the mechanistic constraints contributed not only to predictive performance but also to smoother and more coherent latent regime organization.

**Table 9:** Ablation analysis of the proposed mechanistic-augmented conditional variational autoencoder framework.

Model Configuration	RMSE	MAE	R^2^	VAF (%)	KL Stability	Valid Generated Samples (%)
Standard C-VAE (without mechanistic regularization)	2.41 ± 0.18	1.72 ± 0.14	0.842 ± 0.021	84.6	Moderate	71.2
C-VAE + KL annealing only	2.08 ± 0.15	1.49 ± 0.11	0.873 ± 0.018	87.9	High	79.4
Proposed MCVAE (full model)	1.76 ± 0.11	1.18 ± 0.09	0.914 ± 0.014	91.8	High	89.6
Proposed MCVAE without latent regularization	2.29 ± 0.17	1.61 ± 0.13	0.856 ± 0.020	85.7	Low	74.3

### Validation of cluster-conditioned generated formulations

To evaluate the plausibility of the generated formulation candidates, generated samples were compared against experimentally observed formulation ranges and nearest-neighbor samples within latent space. Only generated candidates remaining within physically realistic particle-size and solubility ranges were retained for analysis. Distances between generated samples and their nearest experimental neighbors were additionally evaluated to assess whether latent sampling remained within experimentally supported formulation regions rather than extrapolating into unrealistic areas. The generated formulations generally preserved coherent multivariate relationships and remained close to experimentally observed formulation neighborhoods.

### Interpretation of the latent formulation structure

The analysis points to the learned latent space functioning more like a continuous formulation behavior manifold than simply a compressed feature embedding. The geometric layout uncovered by the use of dimensionality reduction and probabilistic clustering shows evidence of gradual transitions between formulation conditions, which is totally in line with the idea that changes in the amount of solvent and temperature variations that are part of the process will lead to gradual changes in solubility and particle size. The elements that make up this space do not come from predefined classes but originate from the data together with certain given constraints, so it points to the representation capturing regime continuity that is an inherent feature of formulation science. The solubility property within this order of things seems to be conveyed through several mostly uncorrelated dimensions. There is no singular latent direction that most of the encoding, and a moderate amount of inter-latent associations indicate that the pairs of representations are complementary rather than being redundant. This spread-out encoding makes sense; in fact, it serves as a basis for regime analysis, where the clusters are the result of the latent geometry and not of outside labels imposed on the data. Further examples of how models have behavioral consistency within regimes are given by the latent sampling conditioned on cluster behavior. The generated samples are not only a mere extension of the immediate neighborhood of experimental data, but also, they contain preserved coherent multivariate relationships, which practically means that the space of the latent allows for the structured exploration and not just the memorization of the observed samples. Regime-specific analyses have been quite instrumental in supporting this interpretation. By performing statistical mixture comparisons, it has been revealed that certain areas in the latent space correspond to entirely different particle size, whereas other areas overlap smoothly; thus, the analogy of formulation landscape with gradual boundaries is perfectly fitting. Cluster-conditional PDP patterns also indicate that in different regimes, feature, response relationships change, thus by explaining the dependency on the context of the particle size, the paper accounts for the fact that global interpretations are not always enough when it comes to formulation modeling. Stable training dynamics, the ability to withstand non-Gaussian variable distributions, and distributed SHAP contributions, taken together, point to the internal coherence of the framework. However, the present analysis is limited by the extent of the available formulation data. Nonetheless, the alignment of geometric, statistical, generative, and interpretability types of evidence lends credence to the reliability of the latent formulation structure that has been inferred.

#### Implications for experimental formulation design

**Fig. 11:**
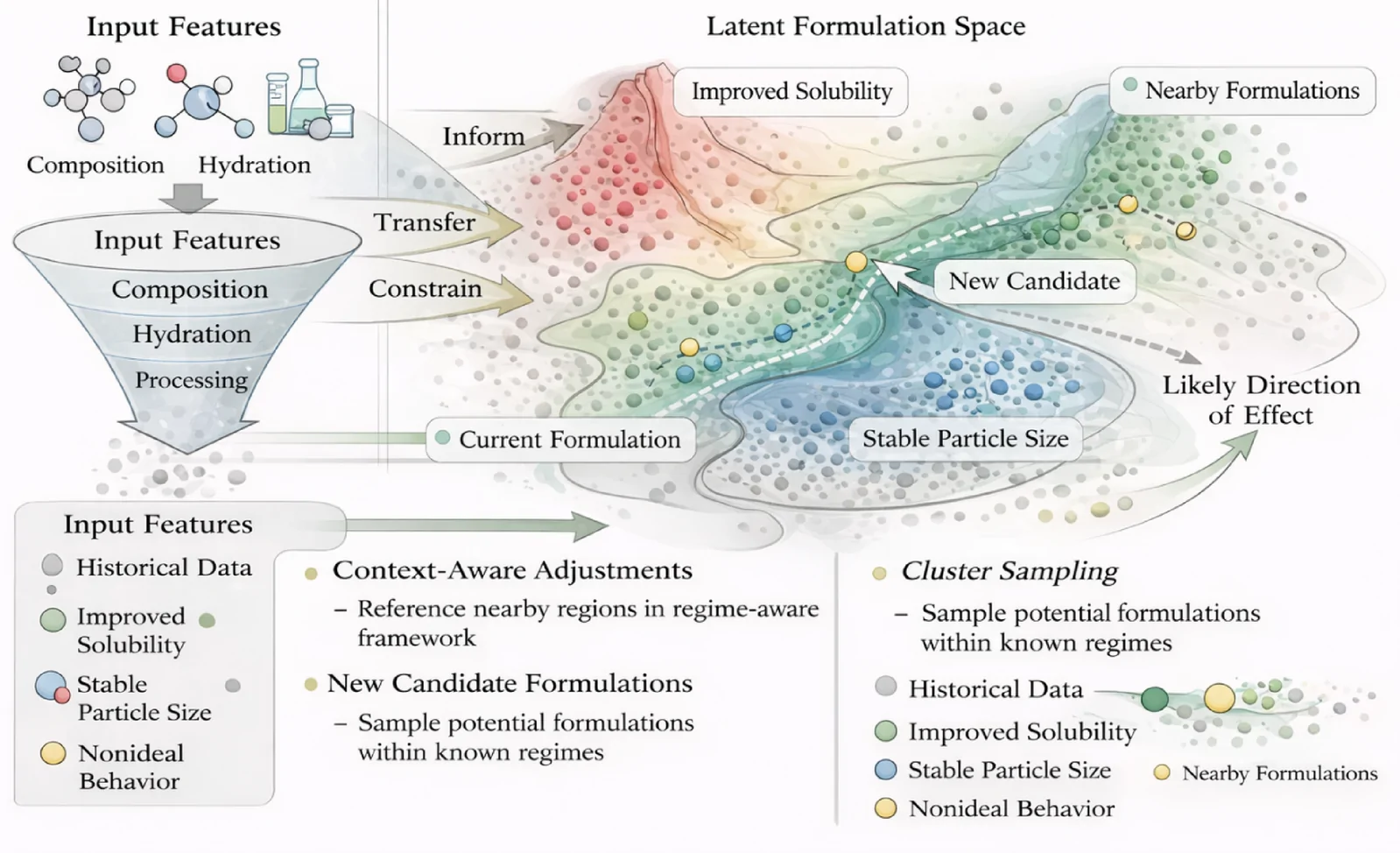
Schematic illustration of how the regime-aware latent formulation space supports context-aware experimental design, enabling informed parameter adjustment, knowledge transfer from historical data, and cluster-conditioned proposal of new formulation candidates. The schematic was created by the authors using Wondershare EdrawMax (Version 14.5.4; https://www.edrawsoft.com/edraw-max/).

In the experimental formulation process, parameter tuning is typically done through iterative trial-and-error. Changes in solvent amount, hydration conditions, or composition are tested experimentally without a clear understanding of the bigger picture. The current framework enables one to think of the formulation space simply as different behavioral regimes of solubility and particle size rather than a set of unrelated samples. Locating a candidate formulation in this regime-aware map provides an opportunity to pre-judge the behavior of nearby formulations in terms of solubility and particle size before making changes to individual parameters. The experiment thus becomes more informed and less of a guesswork tuning. Besides, the findings further clarify a frequently encountered challenge in the laboratory. A variable of the same formulation may appear to cause different outcomes even when the conditions seem to be the same. It demonstrated that feature, response correlations change among latent regimes, and thus the discrepancies in results are not due to experimental errors, but they are manifestations of context-dependent formulation behavior. When what is seen is interpreted using the concept of behavioral regimes, the parameter increase, such as the solvent amount, will lead to different effects on solubility depending on the case. Since molecular descriptors and processing variables are embedded together, the learned representation forms a basis for partial knowledge transfer between compounds and studies. The old formulation data can guide the new experiments by showing which areas of formulation behavior are already known and how similar the configurations behaved. Moreover, the cluster-conditioned latent sampling yields a method of generating plausible formulation candidates that stay within known behavioral regimes while going beyond the previous testing combinations. Thus, pre-experimental exploration and the selection of candidate conditions can be assisted (Fig. 11).

## Conclusion

This study examined whether latent generative modeling can be used not merely for prediction, but for revealing structured formulation behavior patterns governing drug solubility and particle size. The results indicate that such models can organize formulation data into a coherent latent representation where samples are arranged according to shared behavioral characteristics. Rather than serving as a compressed embedding for reconstruction, the learned latent space functions as a continuous landscape through which formulation regimes and their transitions can be explored. Within this landscape, regimes emerge naturally from the organization of formulation variables and physicochemical descriptors, and transitions between them appear gradual rather than categorical. This regime-aware organization is reflected in the non-degenerate geometry of the embedding, where UMAP projections span from − 3.42 to 8.01 and − 3.43 to 10.03 along the two axes, and t-SNE ranges from − 17.07 to − 1.35 and − 6.72 to 5.93. GMM identified seven overlapping components (0–6; average label 3.14), while HDBSCAN classified samples as noise, indicating smooth regime transitions rather than dense separations. Correlations between latent variables and solubility showed distributed encoding across 20 dimensions, with no single dominant axis, consistent with partial disentanglement. The robustness of this interpretation is supported by convergence across independent analyses. Cluster-conditioned latent sampling within a single regime (Cluster 3) generated formulations with predicted solubility ranging from 547.18 to 24,378.54 mg/mL, compared with 249.8 to 1,040 mg/mL for the nearest real samples, while remaining within the same latent region. Wilcoxon tests showed that some cluster pairs differ significantly (e.g., Clusters 1–2, p = 7.6 × 10⁻⁶; 2–5, p = 7.6 × 10⁻⁶), whereas others overlap (e.g., 1–6, p = 0.89), consistent with both separable and continuous formulation changes. PDPs demonstrated regime-specific feature–response patterns, and SHAP analysis confirmed that multiple variables contribute interactively rather than through a single dominant factor. While the analysis remains data-driven and dependent on available formulation studies, the consistent geometric, statistical, generative, and interpretability evidence suggests that formulation behavior can be represented as a structured latent manifold. This provides a coherent basis for examining formulation regimes and supports hypothesis-driven exploration grounded in experimentally observed data. The proposed framework demonstrated the ability to organize coupled formulation behavior governing both solubility-related response characteristics and particle-size variation within a unified latent representation framework. Although the generated formulation candidates demonstrated consistency with experimentally observed latent neighborhoods and physicochemical bounds, prospective laboratory validation was not performed in the current study. Accordingly, the generated samples should be interpreted as computationally plausible formulation hypotheses requiring future experimental verification. Future work should include prospective laboratory synthesis and experimental characterization of generated formulation candidates to evaluate whether the identified latent formulation regimes correspond to experimentally reproducible physicochemical behavior.

## Supplementary Information

Below is the link to the electronic supplementary material.

Supplementary Material 1

Supplementary Material 2

## Data Availability

The dataset and full analysis code used in this study are provided as Supplementary Materials (Supplementary.xlsx and Supplementary Code.pdf) accompanying this article, enabling other researchers to access and reproduce the reported results.

## Abbreviations

ML: Machine learning
SHAP: SHapley Additive exPlanations
VAF: Variance accounted for
UMAP: Uniform manifold approximation and projection
KL: Kullback–Leibler divergence
HDBSCAN: Hierarchical density-based spatial clustering of applications with noise
CVAF: Conditional variance accounted for
t-SNE: T-Distributed stochastic neighbor embedding
PDP: Partial dependence plot
GMM: Gaussian mixture model

## References

[1] Raju, R. V. et al. Space is a latent sequence: A theory of the hippocampus. *Sci. Adv.* **10**, eadm8470 (2024). 39083616 10.1126/sciadv.adm8470 PMC11290523

[2] Ozcan, H. M., Kalkan, S., Yarman-Vural, F. T. L-VAE: Variational Auto-Encoder with Learnable Beta for Disentangled Representation. ArXiv Prepr ArXiv250702619 2025.

[3] Xie, B. et al. Graph-based unsupervised disentangled representation learning via multimodal large language models. *Adv. Neural Inf. Process. Syst.* **37**, 103101–103130 (2024).

[4] Wang, X., Chen, H., Tang, S., Wu, Z. & Zhu, W. Disentangled representation learning. *IEEE Trans. Pattern Anal. Mach. Intell.* **46**, 9677–9696 (2024). 38949944 10.1109/TPAMI.2024.3420937

[5] Uppal, A., Takida, Y., Lai, C. –H., Mitsufuji, Y. Denoising multi-beta vae: Representation learning for disentanglement and generation. ArXiv Prepr ArXiv250706613 2025.

[6] Guo, Z., Li, G., Li, J., Wang, C., Shi, S. Dualvae: Dual disentangled variational autoencoder for recommendation. Proc. 2024 SIAM Int. Conf. Data Min., SIAM; 2024, p. 571–9.

[7] Zhou, X. & Miao, C. Disentangled graph variational auto-encoder for multimodal recommendation with interpretability. *IEEE Trans. Multimed.* **26**, 7543–7554 (2024).

[8] Zhang, Y., Li, J. & Chao, X. ChemNav: An interactive visual tool to navigate in the latent space for chemical molecules discovery. *Vis. Informatics* **8**, 60–70 (2024).

[9] Wang, S., Yang, C. & Chen, L. LSA-DDI: Learning stereochemistry-aware drug interactions via 3D feature fusion and contrastive cross-attention. *Int. J. Mol. Sci.* **26**, 6799. 10.3390/ijms26146799 (2025). 40725046 10.3390/ijms26146799 PMC12294978

[10] Hassan, F. et al. Potential of dietary hemp and cannabinoids to modulate immune response to enhance health and performance in animals: Opportunities and challenges. *Front. Immunol.* **14**, 1285052 (2023). 38111585 10.3389/fimmu.2023.1285052 PMC10726122

[11] Zhang, R., Guo, X., Pan, Y., Gao, S. STAND-Net: A Spiking Temporal Attention autoeNcoDer Network for Efficient EEG Artifact Removal. *IEEE J Biomed Heal Informatics* (2026). 10.1109/JBHI.2026.3670141 41779656

[12] Boyar, O. & Takeuchi, I. Latent space bayesian optimization with latent data augmentation for enhanced exploration. *Neural Comput.* **36**, 2446–2478 (2024). 39312498 10.1162/neco_a_01708

[13] Meng, D. & Liu, Z. Machine learning aided pharmaceutical engineering: Model development and validation for estimation of drug solubility in green solvent. *J. Mol. Liq.* **392**, 123286 (2023).

[14] Obaidullah, A. J. & Mahdi, W. A. Machine learning-based analysis on pharmaceutical compounds interaction with polymer to estimate drug solubility in formulations. *Sci. Rep.* **15**, 23683 (2025). 40603913 10.1038/s41598-025-05535-7 PMC12222824

[15] Li, K. et al. Advances in metal ion–mediated macromolecular drug delivery. *Int. J. Pharm.*10.1016/j.ijpharm.2025.126501 (2025). 41407272 10.1016/j.ijpharm.2025.126501

[16] Schmitt, J. M., Baumann, J. M. & Morgen, M. M. Predicting spray dried dispersion particle size via machine learning regression methods. *Pharm. Res.* **39**, 3223 (2022). 35986124 10.1007/s11095-022-03370-3 PMC9780133

[17] Dey, H., Arya, N., Mathur, H., Chatterjee, N. & Jadon, R. Exploring the role of artificial intelligence and machine learning in pharmaceutical formulation design. *Int. J. Newgen. Res. Pharm. Healthc.* **2**, 30–41 (2024).

[18] Ochiai, T. et al. Variational autoencoder-based chemical latent space for large molecular structures with 3D complexity. *Commun. Chem.* **6**, 249 (2023). 37973971 10.1038/s42004-023-01054-6 PMC10654724

[19] Janela, T., Takeuchi, K. & Bajorath, J. Predicting potent compounds using a conditional variational autoencoder based upon a new structure–potency fingerprint. *Biomolecules* **13**, 393 (2023). 36830761 10.3390/biom13020393 PMC9953226

[20] Fan, X. et al. ICVAE: Interpretable conditional variational autoencoder for de novo molecular design. *Int. J. Mol. Sci.* **26**, 3980 (2025). 40362221 10.3390/ijms26093980 PMC12071458

[21] Ghanavati, M. A., Ahmadi, S. & Rohani, S. A machine learning approach for the prediction of aqueous solubility of pharmaceuticals: A comparative model and dataset analysis. *Digit. Discov.* **3**, 2085–2104 (2024).

[22] Bao, Z. et al. Towards the prediction of drug solubility in binary solvent mixtures at various temperatures using machine learning. *J. Cheminform.* **16**, 117 (2024). 39468626 10.1186/s13321-024-00911-3 PMC11520512

[23] Amiri, M. & Khaleseh, F. Predicting drug solubility in binary solvent mixtures using graph convolutional networks: A comprehensive deep learning approach. *Sci. Rep.*10.1038/s41598-025-28272-3 (2025). 41318549 10.1038/s41598-025-28272-3 PMC12753786

[24] Zeng, C., Khan, Z., Post, N. L., Data-efficient and interpretable inverse materials design using a disentangled variational autoencoder. ArXiv Prepr ArXiv240906740 2024.

[25] Fan, T., Cutforth, M., D’Elia, M., Cortiella, A., Doostan, A., Darve, E. Physically Interpretable Representation Learning with Gaussian Mixture Variational AutoEncoder (GM-VAE). ArXiv Prepr ArXiv251121883 2025.

[26] Hasankhani, M. S. Enhancing Drug Discovery: Autoencoder-Based Latent Space Augmentation for Improved Molecular Solubility Prediction using LatMixSol. ArXiv Prepr ArXiv250600223 2025.

[27] Bhadwal, A. S., Kumari, M. & Kumar, A. PCF-VAE: Posterior collapse free variational autoencoder for de novo drug design. *Sci. Rep.* **15**, 34152 (2025). 41034259 10.1038/s41598-025-14285-5 PMC12488873

[28] Shahiwala, A. F., Qawoogha, S. S. & Faruqui, N. Designing optimum drug delivery systems using machine learning approaches: A prototype study of niosomes. *AAPS PharmSciTech* **24**, 94 (2023). 37012582 10.1208/s12249-023-02547-2

[29] Arafa, M. G. & Ayoub, B. M. DOE optimization of nano-based carrier of pregabalin as hydrogel: New therapeutic & chemometric approaches for controlled drug delivery systems. *Sci. Rep.* **7**, 41503 (2017). 28134262 10.1038/srep41503 PMC5278417

[30] Nasr, M., Mansour, S., Mortada, N. D. & Elshamy, A. A. Vesicular aceclofenac systems: A comparative study between liposomes and niosomes. *J. Microencapsul.* **25**, 499–512 (2008). 18608811 10.1080/02652040802055411

[31] Samifara, S., Akki, R., Ramya, M. G. & Naik, V. V. Development and characterization of carbamazepine loaded nonionic surfactant vesicles. *Acta Chim. Pharm. Indica* **5**, 29–40 (2015).

[32] Sankhyan, A. & Pawar, P. K. Metformin loaded non-ionic surfactant vesicles: Optimization of formulation, effect of process variables and characterization. *DARU J. Pharm. Sci.* **21**, 7 (2013). 10.1186/2008-2231-21-7 PMC3556082 23351604

[33] Bhattacharya, S. Preparation and evaluation of diclofenac sodium niosomes using round bottom flask method. *Asian J. Pharm.*10.22377/ajp.v14i2.3613 (2020).

[34] Akbarzadeh, I. et al. Niosomal delivery of simvastatin to MDA-MB-231 cancer cells. *Drug Dev. Ind. Pharm.* **46**, 1535–1549 (2020). 32808813 10.1080/03639045.2020.1810269

[35] Khalifa, A.-Z.M. & Abdul Rasool, B. K. Optimized mucoadhesive coated niosomes as a sustained oral delivery system of famotidine. *AAPS PharmSciTech* **18**, 3064–3075 (2017). 28516414 10.1208/s12249-017-0780-7

[36] Moghddam, S. R. M., Ahad, A., Aqil, M., Imam, S. S. & Sultana, Y. Formulation and optimization of niosomes for topical diacerein delivery using 3-factor, 3-level Box-Behnken design for the management of psoriasis. *Mater. Sci. Eng. C* **69**, 789–797 (2016). 10.1016/j.msec.2016.07.043 27612773

[37] Ruckmani, K. & Sankar, V. Formulation and optimization of zidovudine niosomes. *AAPS PharmSciTech* **11**, 1119–1127 (2010). 20635228 10.1208/s12249-010-9480-2 PMC2974162

[38] Kamboj, S., Saini, V. & Bala, S. Formulation and characterization of drug loaded nonionic surfactant vesicles (niosomes) for oral bioavailability enhancement. *Sci. World J.* **2014**, 959741 (2014). 10.1155/2014/959741 PMC3929577 24672401

[39] Jadon, P. S., Gajbhiye, V., Jadon, R. S., Gajbhiye, K. R. & Ganesh, N. Enhanced oral bioavailability of griseofulvin via niosomes. *AAPS PharmSciTech* **10**, 1186–1192 (2009). 19856107 10.1208/s12249-009-9325-z PMC2799591

[40] Tamizharasi, S., Dubey, A., Rathi, V. & Rathi, J. C. Development and characterization of niosomal drug delivery of gliclazide. *J. Young Pharm.* **1**, 205 (2009).

[41] Ramadan, A. A., Eladawy, S. A., El-Enin, A. S. M. A. & Hussein, Z. M. Development and investigation of timolol maleate niosomal formulations for the treatment of glaucoma. *J. Pharm. Investig.* **50**, 59–70 (2020).

[42] Teaima, M. H., El Mohamady, A. M., El-Nabarawi, M. A. & Mohamed, A. I. Formulation and evaluation of niosomal vesicles containing ondansetron HCL for trans-mucosal nasal drug delivery. *Drug Dev. Ind. Pharm.* **46**, 751–761 (2020). 32250181 10.1080/03639045.2020.1753061

[43] Shirsand, S. B., Para, M. S., Nagendrakumar, D., Kanani, K. M. & Keerthy, D. Formulation and evaluation of ketoconazole niosomal gel drug delivery system. *Int. J. Pharm. Investig.* **2**, 201 (2012). 23580936 10.4103/2230-973X.107002 PMC3618636

[44] Qumbar, M., Imam, S. S., Ali, J., Ahmad, J. & Ali, A. Formulation and optimization of lacidipine loaded niosomal gel for transdermal delivery: in-vitro characterization and in-vivo activity. *Biomed. Pharmacother.* **93**, 255–266 (2017). 28738502 10.1016/j.biopha.2017.06.043

[45] Das, M. K. & Palei, N. N. Sorbitan ester noisomes for tropical delivery of rofecoxib. *Indian J. Exp. Biol.* **49**, 438 (2011). 21702223

[46] Mueller, T., Kusne, A. G. & Ramprasad, R. Machine learning in materials science: Recent progress and emerging applications. *Rev. Comput. Chem.* **29**, 186–273 (2016).

[47] Noé, F., Tkatchenko, A., Müller, K.-R. & Clementi, C. Machine learning for molecular simulation. *Annu. Rev. Phys. Chem.* **71**, 361–390 (2020). 32092281 10.1146/annurev-physchem-042018-052331

[48] Liu, C. et al. An improved anticancer drug-response prediction based on an ensemble method integrating matrix completion and ridge regression. *Mol. Ther. Nucleic Acids* **21**, 676–686 (2020). 32759058 10.1016/j.omtn.2020.07.003 PMC7403773

[49] Lu, J. Y., Zhang, F. R., Zou, W. Z., Huang, W. T. & Guo, Z. Peptide-based system for sensing Pb2+ and molecular logic computing. *Anal. Biochem.* **630**, 114333 (2021). 34400145 10.1016/j.ab.2021.114333

[50] Zhou, R. et al. NEDD: A network embedding based method for predicting drug-disease associations. *BMC Bioinformatics* **21**, 387 (2020). 32938396 10.1186/s12859-020-03682-4 PMC7495830

[51] Locatello, F., Bauer, S., Lucic, M., Raetsch, G., Gelly, S., Schölkopf, B., et al. Challenging common assumptions in the unsupervised learning of disentangled representations. *Int. Conf. Mach. Learn.*, PMLR; 2019, p. 4114–24.

[52] Goswami, S., Bora, A., Yu, Y. & Karniadakis, G. E. Physics-informed deep neural operator networks. In *Mach. Learn. Model. Simul. Methods Appl.* 219–254 (Springer, 2023).

[53] Meng, Y. et al. Drug repositioning based on weighted local information augmented graph neural network. *Brief. Bioinform.* **25**, bbad431 (2024). 10.1093/bib/bbad431 PMC10686358 38019732

