# Supplementary material for: Discovering interpretable drug formulation behavior patterns via a mechanistic-augmented conditional variational autoencoder
Source: Sci Rep. 2026 Jul 15;16:26033. doi: 10.1038/s41598-026-61464-z (PMC13490609; doi:10.1038/s41598-026-61464-z)
Supplement: Supplementary file 1 [file 41598_2026_61464_MOESM1_ESM.pdf]

```yaml
# ============================================================
# FILE: config.yaml
# ============================================================

# config.yaml

data:
  target_column: Particle size (nm)

preprocessing:
  test_size: 0.3
  random_state: 42

model:
  latent_dim: 20
  beta_max: 2
  kl_anneal_epochs: 50
  lambda_mech: 1.0
  lr: 0.001
  max_epochs: 300
  patience: 20

mechanistic:
  feature_index: 0   # temperature or solvent amount

embedding:
  umap:
```

```
    n_neighbors: 15
    min_dist: 0.1
  tsne:
    perplexity: 30

clustering:
  gmm_k_min: 2
  gmm_k_max: 8
  hdbscan_min_cluster: 30

prediction:
  model: random_forest
  n_estimators: 200

generation:
  n_samples: 114
  target_multiplier: 1.2

interpretability:
  pdp_grid: 20

statistics:
  alpha: 0.05
-e
```


==

```
# FILE: data_loader.py
# ============================================================

# data_loader.py

import pandas as pd
import numpy as np
from sklearn.preprocessing import StandardScaler
from sklearn.model_selection import train_test_split


def load_data(path, target_col, test_size=0.2, random_state=42):
    # =====================
    # Load
    # =====================
    df = pd.read_excel(path)

    if target_col not in df.columns:
        raise ValueError(f"Target column '{target_col}' not found.")

    # =====================
    # Basic validation
    # =====================
    if df.isnull().any().any():
        raise ValueError("Input data contains NaN values.")

    X = df.drop(columns=[target_col]).values
```

```
y = df[target_col].values.reshape(-1, 1)

if not np.issubdtype(X.dtype, np.number):
    raise ValueError("Features must be numeric.")

# =====================
# Train / Validation split
# =====================
X_tr, X_val, y_tr, y_val = train_test_split(
    X, y,
    test_size=test_size,
    random_state=random_state
)

# =====================
# Scaling (NO leakage)
# =====================
sx, sy = StandardScaler(), StandardScaler()

X_tr_s = sx.fit_transform(X_tr)
X_val_s = sx.transform(X_val)

y_tr_s = sy.fit_transform(y_tr)
y_val_s = sy.transform(y_val)

feature_names = df.columns.drop(target_col).tolist()
```

```
    return {
        "X": X,
        "y": y,
        "X_tr": X_tr_s,
        "X_val": X_val_s,
        "y_tr": y_tr_s,
        "y_val": y_val_s,
        "scaler_X": sx,
        "scaler_y": sy,
        "feature_names": feature_names,
        "df_raw": df
    }
-e

# ============================================================
# FILE: model.py
# ============================================================

# model.py

import torch
import torch.nn as nn

device = torch.device("cuda" if torch.cuda.is_available() else "cpu")
```

```python
class Encoder(nn.Module):
    def __init__(self, x_dim, c_dim, z_dim):
        super().__init__()
        self.net = nn.Sequential(
            nn.Linear(x_dim + c_dim, 64),
            nn.ReLU(),
            nn.Linear(64, 32),
            nn.ReLU()
        )
        self.mu = nn.Linear(32, z_dim)
        self.logvar = nn.Linear(32, z_dim)

    def forward(self, x, c):
        h = self.net(torch.cat([x, c], dim=1))
        return self.mu(h), self.logvar(h)


class Decoder(nn.Module):
    def __init__(self, z_dim, c_dim, x_dim):
        super().__init__()
        self.net = nn.Sequential(
            nn.Linear(z_dim + c_dim, 32),
            nn.ReLU(),
            nn.Linear(32, 64),
            nn.ReLU(),
            nn.Linear(64, x_dim)
        )
```

```
    def forward(self, z, c):
        return self.net(torch.cat([z, c], dim=1))


class Predictor(nn.Module):
    """Predict target y from latent z"""
    def __init__(self, z_dim, y_dim):
        super().__init__()
        self.net = nn.Sequential(
            nn.Linear(z_dim, 16),
            nn.ReLU(),
            nn.Linear(16, y_dim)
        )

    def forward(self, z):
        return self.net(z)


class CVAE(nn.Module):
    def __init__(self, x_dim, c_dim, z_dim, y_dim=1):
        super().__init__()
        self.encoder = Encoder(x_dim, c_dim, z_dim)
        self.decoder = Decoder(z_dim, c_dim, x_dim)
        self.predictor = Predictor(z_dim, y_dim)

    def reparameterize(self, mu, logvar):
```

```
        std = torch.exp(0.5 * logvar)
        eps = torch.randn_like(std)
        return mu + eps * std

    def forward(self, x, c, y_dummy=None):
        # اگر y_dummy برای predictor
        if y_dummy is None:
            y_dummy = torch.zeros((x.size(0), 1)).to(x.device)

        mu, logvar = self.encoder(x, c)
        z = self.reparameterize(mu, logvar)
        x_hat = self.decoder(z, c)
        y_hat = self.predictor(z)
        return x_hat, y_hat, mu, logvar, z

    def encode(self, x, c):
        mu, logvar = self.encoder(x, c)
        return self.reparameterize(mu, logvar)

    def predict(self, x, c):
        z = self.encode(x, c)
        return self.predictor(z)
```

-e

```
# ==============================================================
# FILE: train.py
# ==============================================================

# train.py

import torch
import torch.nn as nn
import torch.optim as optim
import numpy as np
import pandas as pd
from model import CVAE, device


def train_cvae(data, cfg):
    # ====================
    # Unpack config
    # ====================
    latent_dim = cfg["model"]["latent_dim"]
    beta_max = cfg["model"]["beta_max"]
    lambda_mech = cfg["model"]["lambda_mech"]
    lr = cfg["model"]["lr"]
    max_epochs = cfg["model"]["max_epochs"]
    patience = cfg["model"]["patience"]

    # ====================
    # Data
```

```
# ====================
X = data["X"]
Xtr = torch.tensor(data["X_tr"], dtype=torch.float32).to(device)
ytr = torch.tensor(data["y_tr"], dtype=torch.float32).to(device)
Xval = torch.tensor(data["X_val"], dtype=torch.float32).to(device)
yval = torch.tensor(data["y_val"], dtype=torch.float32).to(device)


Ctr = torch.tensor(data["y_tr"], dtype=torch.float32, device=device, requires_grad=True)
Cval = torch.tensor(data["y_val"], dtype=torch.float32, device=device, requires_grad=True)

# ====================
# Model
# ====================
model = CVAE(x_dim=X.shape[1], c_dim=1, z_dim=latent_dim).to(device)

optimizer = optim.Adam(model.parameters(), lr=lr)
mse = nn.MSELoss()

# ====================
# Training
# ====================
best_loss = np.inf
wait = 0
history = []

for epoch in range(max_epochs):
```

```
model.train()

beta = min(beta_max, epoch / (0.3 * max_epochs))

optimizer.zero_grad()

x_hat, y_hat, mu, logvar, z = model(Xtr, Ctr)

recon_x = mse(x_hat, Xtr)
recon_y = mse(y_hat, ytr)

kl = -0.5 * torch.mean(1 + logvar - mu.pow(2) - logvar.exp())

# Mechanistic smoothness on y_hat wrt condition
grad = torch.autograd.grad(x_hat.sum(), Ctr, create_graph=True)[0]
mech = torch.mean(grad ** 2)

loss = recon_x + recon_y + beta * kl + lambda_mech * mech
loss.backward()
optimizer.step()

# ====================
# Validation
# ====================
model.eval()
with torch.no_grad():
    xh_v, yh_v, mu_v, lv_v, _ = model(Xval, yval, Cval)
```

```
        val_loss = (
            mse(xh_v, Xval)
            + mse(yh_v, yval)
            + beta * (-0.5 * torch.mean(1 + lv_v - mu_v.pow(2) - lv_v.exp()))
        )

    history.append([
        epoch,
        loss.item(),
        val_loss.item(),
        recon_x.item(),
        recon_y.item(),
        kl.item(),
        mech.item()
    ])

    if val_loss < best_loss:
        best_loss = val_loss
        wait = 0
        torch.save(model.state_dict(), "models/cvae.pt")
    else:
        wait += 1
        if wait >= patience:
            print("■ Early stopping")
            break

# ======================
```

```
    # Save history
    # =====================
    hist_df = pd.DataFrame(
        history,
        columns=[
            "epoch", "train_loss", "val_loss",
            "recon_x", "recon_y", "kl", "mech"
        ]
    )
    hist_df.to_excel("results/training_history.xlsx", index=False)

    print("✅ CVAE training completed.")

    return model
-e


# ============================================================
# FILE: latent.py
# ============================================================

# latent.py

import pandas as pd
import torch

def extract_latent(model, X, y, device="cpu"):
```

```
    """
    Extract latent space Z from trained CVAE.
    X: [n_samples, n_features]
    y: [n_samples, 1] target / condition
    """
    model.eval()
    X_t = torch.tensor(X, dtype=torch.float32).to(device)
    c_t = torch.tensor(y, dtype=torch.float32).reshape(-1, 1).to(device)

    with torch.no_grad():
        mu, logvar = model.encoder(X_t, c_t)

    Z = mu.cpu().numpy()

    # Save latent space
    latent_df = pd.DataFrame(Z, columns=[f"Z{i+1}" for i in range(Z.shape[1])])
    latent_df["y"] = y.ravel()
    latent_df.to_excel("results/latent_space.xlsx", index=False)
    print("✅ Latent space saved.")

    return Z
-e

# ============================================================
# FILE: clustering.py
# ============================================================
```

```python
# ========================
# GMM clustering (Primary)
# ========================
def run_gmm(Z, cfg):
    gmm_cfg = cfg.get("clustering", {})
    k_min = gmm_cfg.get("gmm_k_min", 2)
    k_max = gmm_cfg.get("gmm_k_max", 8)

    bic_scores = []
    K_range = range(k_min, k_max + 1)

    for k in K_range:
        gmm = GaussianMixture(
            n_components=k,
            covariance_type="full",
            random_state=42
        )
        gmm.fit(Z)
        bic_scores.append(gmm.bic(Z))

    bic_df = pd.DataFrame({
        "K": list(K_range),
        "BIC": bic_scores
    })
    bic_df.to_excel("results/gmm_bic_scores.xlsx", index=False)
```

```
    best_k = K_range[np.argmin(bic_scores)]
    print(f"✅ Optimal GMM K selected: {best_k}")

    gmm = GaussianMixture(
        n_components=best_k,
        covariance_type="full",
        random_state=42
    )
    gmm_labels = gmm.fit_predict(Z)

    return gmm_labels


# ===================================
# HDBSCAN clustering (Robustness)
# ===================================
def run_hdbscan(Z, cfg):
    hdb_cfg = cfg.get("clustering", {})
    min_cluster_size = hdb_cfg.get("hdbscan_min_cluster", 30)

    clusterer = hdbscan.HDBSCAN(
        min_cluster_size=min_cluster_size,
        min_samples=None,
        metric="euclidean"
    )

    hdb_labels = clusterer.fit_predict(Z)
```

```python
    n_clusters = len(set(hdb_labels)) - (1 if -1 in hdb_labels else 0)
    noise_ratio = np.mean(hdb_labels == -1)

    print(f"✅ HDBSCAN clusters: {n_clusters}")
    print(f"⚠️ Noise ratio: {noise_ratio:.2%}")

    return hdb_labels


# ==========================================
# Merge clustering results and save Excel
# ==========================================
def attach_clusters(df, gmm_labels, hdb_labels):
    df_out = df.copy()
    df_out["cluster_gmm"] = gmm_labels
    df_out["cluster_hdbscan"] = hdb_labels

    df_out.to_excel(
        "results/latent_with_clusters.xlsx",
        index=False
    )

    print("✅ GMM & HDBSCAN clusters saved.")

    return df_out
```

-e

```
# ============================================================
# FILE: prediction.py
# ============================================================

# prediction.py

import numpy as np
from sklearn.ensemble import RandomForestRegressor
from sklearn.metrics import r2_score, mean_squared_error
from sklearn.model_selection import train_test_split


def latent_prediction(Z, y_scaled, scaler_y, latent_df):
    """
    Train regressor on latent space and predict target.
    Saves predictions next to real values.
    """

    # =====================
    # Train / Test split (for metrics only)
    # =====================
    Xz_tr, Xz_te, y_tr, y_te = train_test_split(
        Z, y_scaled, test_size=0.2, random_state=42
    )
```

```
# ======================
# Regressor
# ======================
reg = RandomForestRegressor(
    n_estimators=400,
    max_depth= 200,
    random_state=42
)
reg.fit(Xz_tr, y_tr.ravel())

# ======================
# Metrics
# ======================
y_pred_te = reg.predict(Xz_te)

r2 = r2_score(y_te, y_pred_te)
rmse = np.sqrt(mean_squared_error(y_te, y_pred_te))

print(f"✅ Latent prediction — R2={r2:.3f}, RMSE={rmse:.3f}")

# ======================
# Predict on ALL samples
# ======================
y_pred_all_scaled = reg.predict(Z)
y_pred_all = scaler_y.inverse_transform(
    y_pred_all_scaled.reshape(-1, 1)
```

```
    ).ravel()

    y_real_all = scaler_y.inverse_transform(y_scaled).ravel()

    # =====================
    # Save predictions
    # =====================
    pred_df = latent_df.copy()
    pred_df["y_real"] = y_real_all
    pred_df["y_pred"] = y_pred_all
    pred_df["error"] = y_pred_all - y_real_all

    pred_df.to_excel("results/latent_predictions_full.xlsx", index=False)

    return reg, pred_df
-e


# ============================================================
# FILE: generative.py
# ============================================================

# generative.py

import numpy as np
import pandas as pd
import torch
```

```python
from sklearn.metrics import pairwise_distances
from model import device


def generate_formulations(
    model,
    reg,
    Z,
    X_real,
    y_real,
    scaler_X,
    scaler_y,
    cfg
):
    """
    Cluster-aware generative optimization in latent space
    """

    # =====================
    # Load merged latent + prediction + cluster file
    # =====================
    df = pd.read_excel("results/latent_predictions_full.xlsx")

    if "cluster_gmm" not in df.columns:
        raise ValueError("cluster_gmm not found. Run clustering first.")

    # =====================
```

```
# Select best cluster
# ======================
cluster_stats = df.groupby("cluster_gmm")["y_pred"].mean()
target_cluster = cluster_stats.idxmax()

print(f"🎯 Target cluster selected: {target_cluster}")

Z_cols = [c for c in df.columns if c.startswith("Z")]
Z_cluster = df[df["cluster_gmm"] == target_cluster][Z_cols].values

# ======================
# Sampling in latent space
# ======================
n_samples = cfg["generation"]["n_samples"]
target_multiplier = cfg["generation"]["target_multiplier"]

z_gen = np.random.normal(
    loc=Z_cluster.mean(axis=0),
    scale=Z_cluster.std(axis=0),
    size=(n_samples, Z_cluster.shape[1])
)

# ======================
# Target conditioning
# ======================
y_target_real = df["y_real"].mean() * target_multiplier
y_target_scaled = scaler_y.transform(
```

```
    np.full((n_samples, 1), y_target_real)
)

# =====================
# Decode generated latent
# =====================
z_t = torch.tensor(z_gen, dtype=torch.float32).to(device)
c_t = torch.tensor(y_target_scaled, dtype=torch.float32).to(device)

model.eval()
with torch.no_grad():
    x_gen_scaled = model.decoder(z_t, c_t).cpu().numpy()

x_gen_real = scaler_X.inverse_transform(x_gen_scaled)

# =====================
# Predict generated y
# =====================
y_gen_scaled = reg.predict(z_gen)
y_gen_real = scaler_y.inverse_transform(
    y_gen_scaled.reshape(-1, 1)
).ravel()

# =====================
# Nearest real sample comparison
# =====================
dist = pairwise_distances(x_gen_real, X_real)
```

```python
nearest_idx = dist.argmin(axis=1)
y_nearest_real = y_real[nearest_idx].ravel()

# =====================
# Save results
# =====================
gen_df = pd.DataFrame(
    x_gen_real,
    columns=[f"X{i+1}" for i in range(x_gen_real.shape[1])]
)

gen_df["y_pred_generated"] = y_gen_real
gen_df["y_real_nearest"] = y_nearest_real
gen_df["target_cluster"] = target_cluster
gen_df["y_target_condition"] = y_target_real

gen_df.to_excel(
    "results/generated_formulations_with_prediction.xlsx",
    index=False
)

print("✅ Generative optimization results saved.")

return gen_df
```

-e

```
# ============================================================
# FILE: interpretability.py
# ============================================================

# interpretability.py

import pandas as pd
from sklearn.ensemble import RandomForestRegressor
from sklearn.inspection import partial_dependence


def run_pdp(df, feature_cols, cfg=None):
    """
    Compute Partial Dependence Plots (PDP) per GMM cluster
    using a surrogate regressor trained on y_pred.
    """

    if "cluster_gmm" not in df.columns:
        raise ValueError("cluster_gmm not found in dataframe.")

    if "y_pred" not in df.columns:
        raise ValueError("y_pred not found in dataframe.")

    clusters = sorted(df["cluster_gmm"].unique())
```

```
pdp_results = []

grid_resolution = 20
if cfg is not None:
    grid_resolution = cfg.get("pdp", {}).get("grid_resolution", 20)

for cl in clusters:
    df_cl = df[df["cluster_gmm"] == cl]

    # skip very small clusters
    if len(df_cl) < 10:
        continue

    X_cl = df_cl[feature_cols].values
    y_cl = df_cl["y_pred"].values  # PDP on surrogate prediction

    surrogate = RandomForestRegressor(
        n_estimators=300,
        random_state=42
    )
    surrogate.fit(X_cl, y_cl)

    for i, feat in enumerate(feature_cols):
        pdp = partial_dependence(
            surrogate,
            X_cl,
            features=[i],
```

```
            grid_resolution=grid_resolution
        )

        values = pdp["values"][0]
        averages = pdp["average"][0]

        for v, a in zip(values, averages):
            pdp_results.append({
                "cluster": cl,
                "feature": feat,
                "feature_value": float(v),
                "pdp_response": float(a)
            })

    pdp_df = pd.DataFrame(pdp_results)
    pdp_df.to_excel("results/pdp_per_cluster.xlsx", index=False)

    print("✅ PDP per cluster saved.")

    return pdp_df
-e


# ==================================================================
# FILE: stats_analysis.py
# ==================================================================
```

```
# statistics.py

import pandas as pd
from scipy.stats import wilcoxon


def wilcoxon_tests(df, min_cluster_size=10):
    """
    Pairwise Wilcoxon signed-rank tests between GMM clusters
    for both y_real and y_pred.
    """

    if "cluster_gmm" not in df.columns:
        raise ValueError("cluster_gmm not found in dataframe.")

    clusters = sorted(df["cluster_gmm"].unique())

    # ========================
    # GT (y_real)
    # ========================
    wilcoxon_results = []

    for i in range(len(clusters)):
        for j in range(i + 1, len(clusters)):
            c1, c2 = clusters[i], clusters[j]

            y1 = df[df["cluster_gmm"] == c1]["y_real"]
```

```python
        y2 = df[df["cluster_gmm"] == c2]["y_real"]

        if len(y1) < min_cluster_size or len(y2) < min_cluster_size:
            continue

        min_len = min(len(y1), len(y2))
        y1_s = y1.sample(min_len, random_state=42)
        y2_s = y2.sample(min_len, random_state=42)

        stat, pval = wilcoxon(y1_s.values, y2_s.values)

        wilcoxon_results.append({
            "cluster_1": c1,
            "cluster_2": c2,
            "n_samples": min_len,
            "wilcoxon_stat": stat,
            "p_value": pval
        })

wilcoxon_df = pd.DataFrame(wilcoxon_results)
wilcoxon_df.to_excel(
    "results/wilcoxon_intercluster.xlsx",
    index=False
)

print("✅ Wilcoxon (y_real) saved.")
```

```
# =========================
# Prediction (y_pred)
# =========================
wilcoxon_pred_results = []

for i in range(len(clusters)):
    for j in range(i + 1, len(clusters)):
        c1, c2 = clusters[i], clusters[j]

        y1 = df[df["cluster_gmm"] == c1]["y_pred"]
        y2 = df[df["cluster_gmm"] == c2]["y_pred"]

        if len(y1) < min_cluster_size or len(y2) < min_cluster_size:
            continue

        min_len = min(len(y1), len(y2))
        y1_s = y1.sample(min_len, random_state=42)
        y2_s = y2.sample(min_len, random_state=42)

        stat, pval = wilcoxon(y1_s.values, y2_s.values)

        wilcoxon_pred_results.append({
            "cluster_1": c1,
            "cluster_2": c2,
            "n_samples": min_len,
            "wilcoxon_stat": stat,
            "p_value": pval
```

```
        })

    wilcoxon_pred_df = pd.DataFrame(wilcoxon_pred_results)
    wilcoxon_pred_df.to_excel(
        "results/wilcoxon_intercluster_prediction.xlsx",
        index=False
    )

    print("✅ Wilcoxon (prediction) saved.")

    return wilcoxon_df, wilcoxon_pred_df

-e


# ============================================================
# FILE: umap_tsne.py
# ============================================================

# umap_tsne.py

import pandas as pd
import umap
from sklearn.manifold import TSNE
import matplotlib.pyplot as plt
import os
```

```
def run_umap_tsne(
    latent_file="results/latent_predictions_full.xlsx",
    out_dir="results",
    random_state=42
):
    """
    Run UMAP and t-SNE on latent space and save:
    - 2D embeddings (Excel)
    - Visualization plots (PNG)
    """

    os.makedirs(out_dir, exist_ok=True)

    df = pd.read_excel(latent_file)

    # ---------------------
    # Detect latent columns
    # ---------------------
    z_cols = [c for c in df.columns if c.startswith("Z")]
    if len(z_cols) < 2:
        raise ValueError("Latent dimension < 2. UMAP/t-SNE not possible.")

    Z = df[z_cols].values

    # ---------------------
    # UMAP
```

```
# ---------------------
umap_model = umap.UMAP(
    n_components=2,
    n_neighbors=15,
    min_dist=0.1,
    random_state=random_state
)

Z_umap = umap_model.fit_transform(Z)

df_umap = df.copy()
df_umap["UMAP_1"] = Z_umap[:, 0]
df_umap["UMAP_2"] = Z_umap[:, 1]

df_umap.to_excel(
    os.path.join(out_dir, "latent_umap_2d.xlsx"),
    index=False
)

# ---------------------
# t-SNE (sensitivity)
# ---------------------
tsne_model = TSNE(
    n_components=2,
    perplexity=30,
    learning_rate="auto",
    init="pca",
```

```
    random_state=random_state
)

Z_tsne = tsne_model.fit_transform(Z)

df_tsne = df.copy()
df_tsne["TSNE_1"] = Z_tsne[:, 0]
df_tsne["TSNE_2"] = Z_tsne[:, 1]

df_tsne.to_excel(
    os.path.join(out_dir, "latent_tsne_2d.xlsx"),
    index=False
)

# ---------------------
# Plot helper
# ---------------------
def scatter_plot(x, y, c, title, fname, cmap="viridis"):
    plt.figure(figsize=(6, 5))
    sc = plt.scatter(x, y, c=c, cmap=cmap, s=20, alpha=0.85)
    plt.colorbar(sc)
    plt.title(title)
    plt.tight_layout()
    plt.savefig(fname, dpi=300)
    plt.close()

# ---------------------
```

```
# UMAP plots
# ---------------------
if "cluster_gmm" in df_umap.columns:
    scatter_plot(
        df_umap["UMAP_1"],
        df_umap["UMAP_2"],
        df_umap["cluster_gmm"],
        "UMAP – colored by GMM cluster",
        os.path.join(out_dir, "umap_by_cluster.png"),
        cmap="tab10"
    )

if "y_real" in df_umap.columns:
    scatter_plot(
        df_umap["UMAP_1"],
        df_umap["UMAP_2"],
        df_umap["y_real"],
        "UMAP – colored by y_real",
        os.path.join(out_dir, "umap_by_y_real.png")
    )

if "y_pred" in df_umap.columns:
    scatter_plot(
        df_umap["UMAP_1"],
        df_umap["UMAP_2"],
        df_umap["y_pred"],
        "UMAP – colored by y_pred",
```

```
        os.path.join(out_dir, "umap_by_y_pred.png")
    )

# ---------------------
# t-SNE plots
# ---------------------
if "cluster_gmm" in df_tsne.columns:
    scatter_plot(
        df_tsne["TSNE_1"],
        df_tsne["TSNE_2"],
        df_tsne["cluster_gmm"],
        "t-SNE – colored by GMM cluster",
        os.path.join(out_dir, "tsne_by_cluster.png"),
        cmap="tab10"
    )

if "y_real" in df_tsne.columns:
    scatter_plot(
        df_tsne["TSNE_1"],
        df_tsne["TSNE_2"],
        df_tsne["y_real"],
        "t-SNE – colored by y_real",
        os.path.join(out_dir, "tsne_by_y_real.png")
    )

if "y_pred" in df_tsne.columns:
    scatter_plot(
```

```
            df_tsne["TSNE_1"],
            df_tsne["TSNE_2"],
            df_tsne["y_pred"],
            "t-SNE – colored by y_pred",
            os.path.join(out_dir, "tsne_by_y_pred.png")
        )

    print("✅ UMAP & t-SNE embeddings and plots saved.")


if __name__ == "__main__":
    run_umap_tsne()
-e


# ==========================================================
# FILE: run_pipeline.py
# ==========================================================

# main.py

import yaml
import pandas as pd
import numpy as np

from data_loader import load_data
from model import CVAE, device
```

```python
from train import train_cvae
from latent import extract_latent
from clustering import run_gmm, run_hdbscan, attach_clusters
from prediction import latent_prediction
from generative import generate_formulations
from interpretability import run_pdp
from stats_analysis import wilcoxon_tests
from umap_tsne import run_umap_tsne

# -------------------------------
# Load config
# -------------------------------
with open("config.yaml", "r") as f:
    cfg = yaml.safe_load(f)

target_col = cfg["data"]["target_column"]

# -------------------------------
# Load data
# -------------------------------
data_file = "data/Drug Delivery 2.xlsx"
data = load_data(data_file, target_col)

X = data["X"]
y = data["y"]
X_tr = data["X_tr"]
X_val = data["X_val"]
```

```
y_tr = data["y_tr"]
y_val = data["y_val"]
scaler_X = data["scaler_X"]
scaler_y = data["scaler_y"]
feature_names = data["feature_names"]
df_raw = data["df_raw"]

# -------------------------------
# Initialize model
# -------------------------------
LATENT_DIM = cfg["model"]["latent_dim"]
model = CVAE(x_dim=X.shape[1], c_dim=1, z_dim=LATENT_DIM).to(device)


# -------------------------------
# STEP 1: Train CVAE
# -------------------------------
train_cvae(data, cfg)

# -------------------------------
# STEP 2: Extract Latent Space
# -------------------------------
Z = extract_latent(
    model,
    np.vstack([data["X_tr"], data["X_val"]]),
    np.vstack([data["y_tr"], data["y_val"]]),
    device
```

```
)

# -------------------------------
# STEP 3: Clustering
# -------------------------------
gmm_labels = run_gmm(Z, cfg)
hdb_labels = run_hdbscan(Z, cfg)

df_latent = pd.read_excel("results/latent_space.xlsx")
df_latent = attach_clusters(df_latent, gmm_labels, hdb_labels)

# -------------------------------
# STEP 4: Latent Prediction
# -------------------------------
latent_df = pd.read_excel("results/latent_space.xlsx")
reg, df_latent = latent_prediction(Z, np.vstack([data["y_tr"], data["y_val"]]), data["scaler_y"],
df_latent)

df_latent.to_excel("results/latent_predictions_full.xlsx", index=False)

feature_cols = data["feature_names"]
df_latent[feature_cols] = pd.DataFrame(data["X"], columns=feature_cols)

# -------------------------------
# STEP 5: Generative Optimization
# -------------------------------
generate_formulations(model, reg, Z, data["X"], data["y"], data["scaler_X"], data["scaler_y"],
cfg)
```

```
# -------------------------------
# STEP 6: Interpretability (PDP)
# -------------------------------
run_pdp(df_latent, data["feature_names"])

# -------------------------------
# STEP 7: Statistics (Wilcoxon)
# -------------------------------
wilcoxon_tests(df_latent)

# -------------------------------
# STEP 8: UMAP + t-SNE visualization
# -------------------------------
run_umap_tsne(latent_file="results/latent_predictions_full.xlsx")
-e
```
